# DeepQA: A Unified Transcriptome‐Based Aging Clock Using Deep Neural Networks

**DOI:** 10.1111/acel.14471

**Published:** 2025-01-05

**Authors:** Hongqian Qi, Hongchen Zhao, Enyi Li, Xinyi Lu, Ningbo Yu, Jinchao Liu, Jianda Han

**Affiliations:** ^1^ State Key Laboratory of Medicinal Chemical Biology Nankai University Tianjin China; ^2^ College of Pharmacy Nankai University Tianjin China; ^3^ College of Artificial Intelligence Nankai University Tianjin China; ^4^ Engineering Research Center of Trusted Behavior Intelligence, Ministry of Education Nankai University China

## Abstract

Understanding the complex biological process of aging is of great value, especially as it can help develop therapeutics to prolong healthy life. Predicting biological age from gene expression data has shown to be an effective means to quantify aging of a subject, and to identify molecular and cellular biomarkers of aging. A typical approach for estimating biological age, adopted by almost all existing aging clocks, is to train machine learning models only on healthy subjects, but to infer on both healthy and unhealthy subjects. However, the inherent bias in this approach results in inaccurate biological age as shown in this study. Moreover, almost all existing transcriptome‐based aging clocks were built around an inefficient procedure of gene selection followed by conventional machine learning models such as elastic nets, linear discriminant analysis etc. To address these limitations, we proposed DeepQA, a unified aging clock based on mixture of experts. Unlike existing methods, DeepQA is equipped with a specially designed Hinge‐Mean‐Absolute‐Error (Hinge‐MAE) loss so that it can train on both healthy and unhealthy subjects of multiple cohorts to reduce the bias of inferring biological age of unhealthy subjects. Our experiments showed that DeepQA significantly outperformed existing methods for biological age estimation on both healthy and unhealthy subjects. In addition, our method avoids the inefficient exhaustive search of genes, and provides a novel means to identify genes activated in aging prediction, alternative to such as differential gene expression analysis.

## Graphical abstract

DeepQA, a unified aging clock based on mixture of experts which is trained on both healthy and unhealthy subjects.

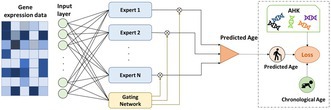

## 1 Introduction

Biological aging is a very complex process and driven by/related to many cellular and biological processes. Many efforts have been made to understand biological aging and to tackle aging‐related diseases. In recent years, thanks to high‐throughput sequencing techniques, large‐scale omics data are now available and provide new opportunities for gaining a deeper understanding of biological aging.

Predicting biological age of a subject using machine learning methods, colloquially termed aging clocks, is an important means/task to understand the complex biological process of aging and to facilitate clinical decision‐making. A widely accepted hypothesis is that the estimated age of aging clocks may reflect the aging rate of an individual to some extent, thus can serve as a measure of biological age (Rutledge, Oh, and Wyss‐Coray 2022).

Built upon different types of omics data, aging clocks can be categorized into different categories, such as DNA methylation aging clocks (Bocklandt et al. 2011; Horvath 2013; Hannum et al. 2013; Zhang et al. 2017), proteomic aging clocks (Pappireddi, Martin, and Wühr 2019; Suhre, McCarthy, and Schwenk 2021), and transcriptomic aging clocks (López‐Otín et al. 2023; Fleischer et al. 2018; Shokhirev and Johnson 2021; Meyer and Schumacher 2021). For instance, Hannum et al. proposed an elastic net‐based DNA methylation aging clock trained on methylomic and clinical parameters, such as gender and body mass index (BMI) and achieved a validation error of 4.9 years (root‐mean‐squared‐error, RMSE) (Hannum et al. 2013). Zhang et al. developed a second generation epigenetic clocks where a mortality risk score based on 10 selected CpGs exhibits strong association with all‐cause mortality (Zhang et al. 2017). Though that DNA methylation aging clocks have been found to be highly reproducible, the resulting models are generally very difficult to be interpreted to understand the molecular and cellular causes and consequences of genomic CpG methylation (Rutledge, Oh, and Wyss‐Coray 2022). In contrast, transcriptomic aging clocks try to directly link aging to genes (RNA gene expression levels) and become a promising solution alternative to other types of aging clocks.

In this study, we focus on the development of transcriptomic aging clocks. Prior work of this line of research are such as (López‐Otín et al. 2023; Fleischer et al. 2018; Shokhirev and Johnson 2021; Meyer and Schumacher 2021). Among them, Fleischer et al. uses an ensemble of linear discriminant analysis as age predictor and the genes for the model training were selected with explicit filtering process and obtained a prediction error of 7.7 years (The prediction accuracies of aging clocks in different references may not be directly comparable due to different datasets and evaluation protocols.) (mean‐absolute‐error, MAE) (Fleischer et al. 2018). Shokhirev et al. proposes to use standard differential expression analyses to select top 1000 differential/variable genes and train random forests on the top of these genes to predict ages with a prediction accuracy of 3.22 years (RMSE) (Shokhirev and Johnson 2021). Meyer et al. employed an expensive gene selection along with elastic net as age predictor and achieved a prediction error of 6.63 years (MAE) (Meyer and Schumacher 2021).

Though some of these methods showed promising performance of age prediction, they suffered from several major drawbacks:

Firstly, almost all existing aging clocks follow the protocol of training machine learning models only on healthy subjects to predict their chronological age, but to infer the biological age on both healthy and unhealthy subjects. This protocol seems to be reasonable since we do not have the accurate biological age of unhealthy subjects. However, as the model is only trained on healthy subjects, the deviation of predicted age from the chronological age of an unhealthy subject in the test set could be caused by abnormal aging process of the individual and/or the incompetence of the machine learning model dealing with the domain gap as the model is not trained on unhealthy subjects. As a result, the estimated biological age could be inaccurate. Addressing this problem is challenging and requires novel training protocol for unhealthy subjects. In this paper, we propose a simple yet effective training loss, called hinge‐mean‐absolute‐error loss, which allows unhealthy subjects participate in training of the aging clock.

Secondly, the prediction accuracy of these methods is still limited, and many involve iterative gene selection prior to training a prediction model which demands heavy computational resources. For large scale datasets, these approaches of gene selection could be prohibitively expensive to carry out. Moreover, we observed that some of existing work of aging clocks failed to avoid the selection bias in gene selection process (Ambroise and McLachlan 2002). In other words, they did not follow the standard testing protocol which is widely accepted to secure a valid evaluation of generalization ability of methods. Consequently, the prediction performance can be seriously overestimated.

One potential solution to address these limitations is deep learning which has shown to excel in learning complex representations from a broad spectrum of tasks involving different kinds of high‐dimensional data with proper design and training strategies (Noothout et al. 2022; Krizhevsky, Sutskever, and Hinton 2012; Salehinejad et al. 2018; Zhang et al. 2020; Liu et al. 2021; Wu et al. 2024). Especially, deep learning has found many successful applications in biology and healthcare etc. (Korsunsky et al. 2019; Du et al. 2019; Eraslan et al. 2019; Zhao et al. 2021; Ceglia et al. 2023). Recently Mohamadi et al. presents an attempt to apply deep neural networks to human age estimation from gene expression data without gene selection as a prior step. In their approach, the input features (genes) were reshaped to 2D image‐alike signals so that they can be processed by convolutional layers. However, unlike images where neighboring pixels are indeed spatially close to one another, genes, and their neighbors in these image‐alike 2D signals are not necessarily related. This may explain why the performance of this method, as shown in the experimental section, was not entirely satisfactory. This motivates us to explore the feasibility of using deep learning for aging estimation on transcriptomic data.

Moreover, deep learning has shown great potential as prediction models in aging estimation involving various data types (Bao et al. 2023), especially images of X‐rays or MRI etc. For instance, lens photographs and deep learning were used to predict biological age of humans and access the risks of age‐related eye and systemic diseases (Ma et al. 2021; Li et al. 2023). Another example is brain aging estimation which could involve three categories of biomarkers, namely functional, imaging, and body fluid ones. For imaging itself, it includes more than two different kinds of data: functional magnetic resonance imaging (fMRI), optical coherence tomography (OCT) etc. (Mishra, Beheshti, and Khanna 2023; Aging Biomarker Consortium et al. 2023).

Owing to the power of deep models, even more complicated and unconventional types of data can be used as biomarkers to predict the degree of aging. In particular, Savcisens et al. proposed a transformer‐based deep model *life2vec* which takes information about life‐events related to health, education, occupation, income, address and working hours, and predict diverse outcomes ranging from early mortality to personality nuances (Savcisens et al. 2023). This method has shown to significantly outperform state‐of‐the‐art methods.

**FIGURE 1:**
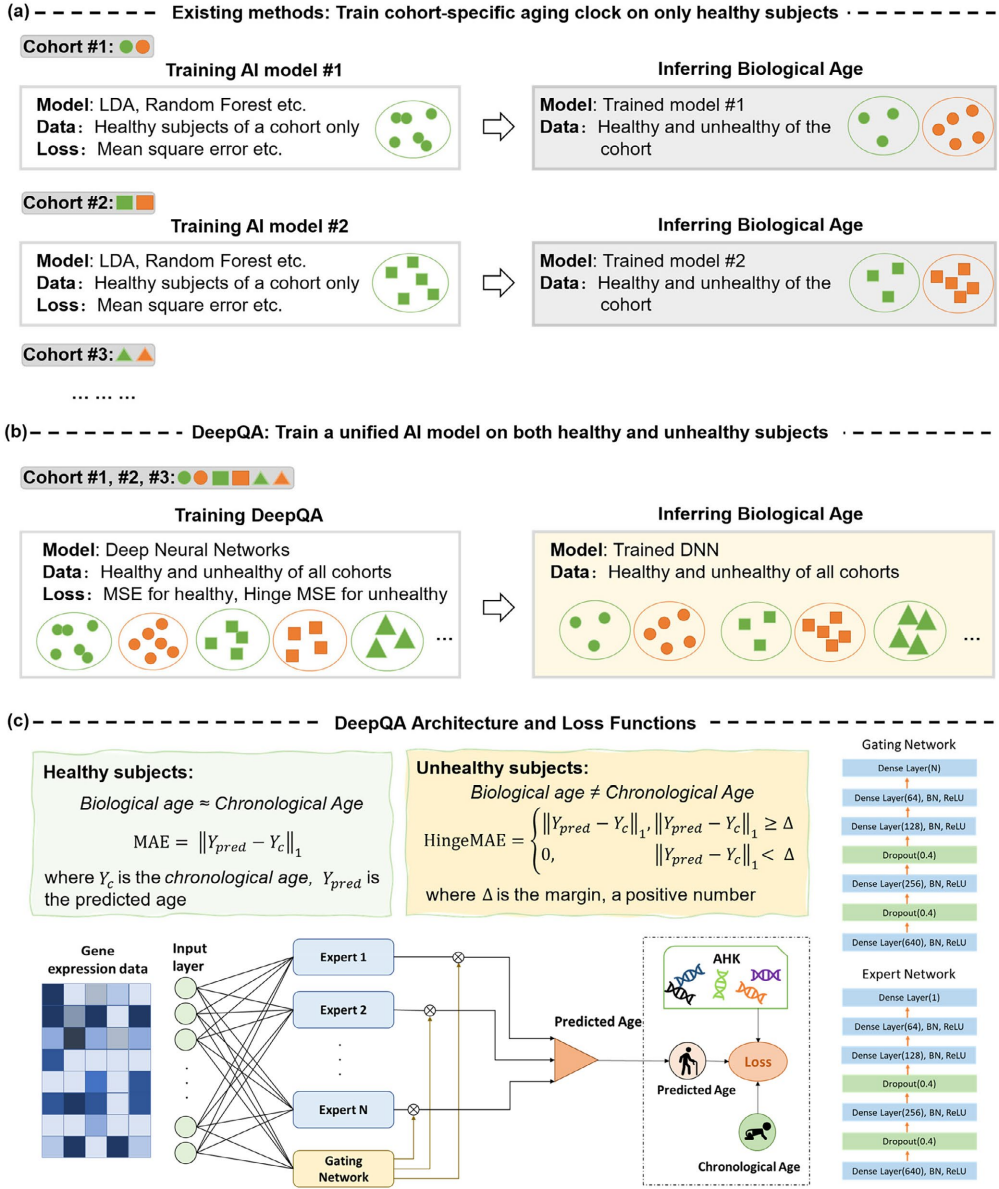
A graphical illustration of the proposed DeepQA compared to existing methods. (a) Existing methods train an AI model for each cohort with homogeneous data as aging clocks to evaluate biological ages. The models, typically random forest, LDA with inefficient gene selection, are usually trained only on healthy/control subjects and tested on both healthy and unhealthy subjects. (b) We propose a unified transcriptome‐based aging clock based on deep neural networks, named DeepQA. Unlike existing methods, DeepQA is trained on data of both healthy and unhealthy subjects on multiple cohorts and is able to infer biological age much more accurate than existing methods. (c) Architecture of the proposed DeepQA as well as specially designed loss functions for the training on both healthy and unhealthy subjects. Our DeepQA has 26.3 M trainable parameters in total.

In this paper, we proposed a transcriptome‐based aging clock for biological age estimation, named as “DeepQA” which stands for *Deep learning for Quantifying Aging*. DeepQA is equipped with a novel loss function, named hinge‐mean‐absolute‐error (Hinge‐MAE) loss, so that it can be trained on unhealthy subjects and estimate biological age more accurately. To the best of our knowledge, this is the first aging clock which trains on both healthy and unhealthy subjects from multiple cohorts. Inside DeepQA, a mixture of expert model (MoE) is employed to model the mapping from the gene expression data to biological ages. DeepQA takes expression data of a full set of genes as inputs and does not need any prior gene selection. Instead, the prominent genes which may be related to aging process are identified through analyzing the saliency map of the trained deep model. DeepQA enjoys the merits of achieving superior performance while avoiding the expensive or even biased gene selection during the process of model fitting. Figure 1 presents a graphical illustration of DeepQA with a comparison to existing methods.

## 2 Materials and Methods

From the machine learning point of view, the difficulty of using deep learning for aging prediction with gene expression data is mainly due to two reasons. Firstly, gene expression data can be regarded as so‐called tabular data and deep neural networks have been experienced difficulties in dealing with tabular data in contrast to signals, for example, images, words and so on (Shwartz‐Ziv and Armon 2022). Secondly, for age estimation, there are often limited number of samples for training compared to the high‐dimensional inputs of genes (> 14 K). This is usually referred to as small data or few shot learning in the machine learning community which has been attracting much attentions. This makes aging prediction using deep learning even harder. Technically, this is also the reason why existing methods for age prediction usually require gene selection prior to learning a predictor, as their ability of handling high‐dimensional data is limited compared to deep learning methods (Rutledge, Oh, and Wyss‐Coray 2022).

One way to tackle these difficulties is to use multiple layer perceptrons (MLPs), also called dense layers, which is suitable for processing unordered gene expression data (Cheng et al. 2024; Agarwal et al. 2021), and to combine multiple cohorts of samples and create a larger database for training. Furthermore, by stacking MLPs to form a Mixture of Experts (MoEs), we are able to train a unified aging clock which predict biological age across diverse types of samples from different tissues, sexes, or health conditions.

### 2.1 DeepQA: Quantifying Aging With Mixture of Experts on Both Healthy and Unhealthy Subjects

Mixture of experts has been widely used in various applications in healthcare, recognition etc. It was originally introduced by (Jacobs et al. 1991) which is an ensemble of several experts/sub‐models controlled by a learnable gate. The gate learns to dispatch each of the inputs only to one of the experts. This architecture makes MoE an excellent learner especially suitable for tasks with heterogeneous data where multiple modes exist (Yuksel, Wilson, and Gader 2012; Chen et al. 2022).

A graphical illustration of the proposed DeepQA is shown in Figure 1. Inside DeepQA, a mixture of experts model is trained to predict biological age given gene expression data as inputs. The loss term for training on healthy subjects is straightforward as their biological age is equal to the chronological age, and we adopt mean‐absolute‐error (MAE) which has been widely used for training aging clocks. However, it is nontrivial to train on unhealthy subjects as their biological age could deviate from their chronological age. We therefore propose a novel loss term, named hinge‐mean‐absolute‐error (Hinge‐MAE), for unhealthy subjects.

Formally, assume that the gene expression data of a sample is denoted as **x**, a 1D vector of length *d* where *d* is the number of genes involved. In each minibatch, DeepQA receives a tensor **X** of shape d×m which is formed by m samples in the current minibatch. Each of the experts $F_{i}\left(\cdot \right)$ takes X as input and makes prediction $Y_{i}$ of 1 × m,

(1) $$Y_{i}=F_{i}\left(X\right)$$

The total loss is then calculated as

(2) $$\begin{array}{c} L_{\text{total}}=\sum_{i}p_{i}L\left(Y,Y_{c}\right)=\sum_{i}p_{i}\left(\lambda D\left(Y_{i},Y_{c}\right)+\gamma L_{\mathrm{AHK}}\right) \end{array}$$

where $p_{i}$ is the output of the gating network to weight the prediction of the $i^{\mathrm{th}}$ expert $F_{i}\left(\cdot \right)$. $Y_{c}$ is the training targets, that is, chronological age. $D\left(\cdot ,\cdot \right)$ is the prediction error. For healthy subjects, it is simply mean‐absolute‐error. For unhealthy subject, it is our proposed Hinge‐MAE which is defined below. $L_{\mathrm{AHK}}$ is the alignment to human knowledge loss. λ and γ are positive weights to balance the loss terms and have been set to 30 and 100, respectively.

(3) $$\begin{array}{c} \text{Hinge}-\mathrm{MAE}=\left\{\begin{array}{c} {\left\|Y_{\text{pred}}-Y_{c}\right\|}_{1},{\left\|Y_{\text{pred}}-Y_{c}\right\|}_{1}\geq ∆\\ 0,{\left\|Y_{\text{pred}}-Y_{c}\right\|}_{1}<∆ \end{array}\right. \end{array}$$

where ∆ is a positive number and serves as a margin to allow the model to output predictions that deviate from the labels, that is, chronological age of unhealthy subjects. In practice, ∆ should be regarded as a hyperparameter and it was set to five in our experiments. The rationale behind this loss is that, the prior knowledge that the biological age of unhealthy subjects could deviate from their chronological age can be modeled as “learning with inaccurate labels,” which is then addressed by designing an explicit margin for the prediction error.

The number of experts is a hyperparameter to tune and has been set to two in our experiments. Each expert contains five dense layers with 640, 256, 128, 64, and 1 neurons, respectively, along with batch normalization and the nonlinear activation function ReLU. Dropout was also applied to counter overfitting (Srivastava et al. 2014). The gating network has the same architecture with the experts except for the head for the output. Overall, the proposed DeepQA has 26.3 M trainable parameters. Detailed architectures of the experts and the gating network can be found in Figure 1c.

### 2.2 Identifying Important Genes via Saliency Map

In prior work, there are three typical ways of selecting genes, among which the most popular one is selecting genes of candidates via, for example, Differential/Variable Expression Analysis (DEA), filtering or through unsupervised learning (Mohamadi and Adjeroh 2021). It is usually computationally efficient than iterative search since no model fitting is involved. However, this strategy is not immune to selection bias if the labels of age are used within the selection process. For instance, when performing DEA, one at least needs to set up two groups, for example, young and old subjects where the information of age goes into the gene selection process. Additional care needs to be taken to avoid selection bias. Secondly, researchers have also proposed to find a good subset of all genes by *iterative/exhaustive search*. Theoretically, it can produce good results but is extremely inefficient as it needs to train a large number of models. For small models, such as LDA, random forest, etc., the computation time is almost unbearable if without a substantial amount of computing resources. Obviously, it is infeasible for large deep learning models. The third way is using the genes that are known to be related to aging in literature. The disadvantage is also obvious, as the overall performance is limited by using only existing human knowledge, especially when some of them may be outdated or questionable.

Saliency map has been a very popular and effective tool to explain/visualize the behaviors of neural networks (Selvaraju et al. 2017). It can be calculated efficiently within the framework of deep learning such as Pytorch. For our application, explaining how DeepQA works is of course valuable, but more importantly, saliency map allows us to infer genes that are important for age estimation. This is much more efficient computationally than traditional approaches such as SHAP (Lundberg and Lee 2017; Hartman et al. 2023).

The saliency map for each expert $S_{F_{i}}$ is basically the partial derivative of its output w.r.t the input and can be calculated as

(4) $$\begin{array}{c} S_{F_{i}}=\left|\frac{\delta F_{i}\left(x\right)}{\delta x}\right| \end{array}$$

Overall, the saliency map can be calculated

(5) $$\begin{array}{c} S_{\text{total}}=\sum_{i}p_{i}S_{F_{i}}=\sum_{i}p_{i}\left|\frac{\delta F_{i}\left(x\right)}{\delta x}\right| \end{array}$$

In practice, this can be easily realized using automatic differentiation in PyTorch.

### 2.3 AHK Loss: Alignment to Human Knowledge

As a powerful data‐driven method, deep neural networks are typically able to learn knowledge purely from the training data. So given enough data, neural networks could learn biological knowledge spontaneously to some extent. However, it is often beneficial to incorporate human knowledge into the architecture of the networks. For applications related to biology or healthcare, these models are often referred to as biologically informed neural networks (Hartman et al. 2023; Fortelny and Bock 2020; Elmarakeby et al. 2021).

For transcriptome‐based aging estimation, datasets are relatively small compared to applications in such as computer vision or natural language processing, it becomes even more critical for deep neural networks to embrace the biological human knowledge accumulated over years. In this study, we propose to align the learning process of DeepQA to the human knowledge by adding an additional loss term, named as *AHK loss*, which measures the similarity between important genes in the current epoch and the genes known to be related to aging in the literature (defined as *aging gene pool* in our work). During training, the AHK loss is combined with other losses, for example, MAE to guide the learning process of the prediction model.

Another motivation of us proposing the AHK loss is *sparsity or gene selection*. Since gene selection does not exist in our method as a preprocessing step, a mechanism of forcing the prediction model to use a subset of important genes such as sparsity constraint would be crucial. In DeepQA, we do not use sparsity constraint such as $L_{1}$ regularization as its shrinking direction is controlled purely by optimizing the age prediction error and could be biased to a wrong set of genes. Instead, we designed the AHK loss which can serve as a sparsity constraint or a soft version of “gene selection.” Imaging an extreme case of using only the AHK loss, the prediction would be carried out only with a small set of known genes which are indubitably sparse.

We decide a set of genes, referred to as *Aging Gene Pool*, which are known to be related to aging in the literature. We denote this pool as $G^{⋆}=\left(g_{1}^{⋆},\ldots ,g_{n}^{⋆}\right)$. For every epoch during training, we compute the saliency map (gene importance) and produce a list of genes sorted by their importance, denoted as $G^{\left(t\right)}=\left(g_{1}^{\left(t\right)},\ldots ,g_{k}^{\left(t\right)}\right)$. The AHK loss is given by

(6) $$\begin{array}{c} L_{\mathrm{AHK}}=-\frac{\sum_{g^{\left(t\right)}\in G^{⋆}}S\left(g^{\left(t\right)}\right)}{\sum_{g^{\left(t\right)}}S\left(g^{\left(t\right)}\right)} \end{array}$$

Obviously, $L_{\mathrm{AHK}}\in \left[-1,0\right]$. We minimize this loss to encourage the model to use genes in the aging gene pool G to make prediction. When only genes in G are used for prediction, $L_{\mathrm{AHK}}=-1$. When none in G is used, $L_{\mathrm{AHK}}=0$.

In our implementation, we compiled N=646 genes which are known to be related to aging as the aging gene pool (Mao et al. 2023). In practice, it can be chosen as any genes that serve the users' applications. Particularly in our experiments, we chose n=10%×N of total genes randomly to compute the AHK loss. n should be treated as a hyperparameter.

### 2.4 Simulating Inherent Noises in Gene Expression Data Using Principal Component Analysis

To examine the robustness of aging clocks against inherent noises in the gene expression data, we employed a classic technique principal component analysis (PCA) to find factors which may act as sources of noises (to be exact, small variations) (Bishop 2006; Manjón, Coupé, and Buades 2015). We simply will refer to this kind of noises as PCA noises. By manipulating these factors, we can simulate inherent noises which are used to test the robustness of an aging clock.

Formally, any (normalized) sample of gene expression data can be decomposed into a linear weighted combination of principal components (PCs, which are learned from the data),

(7) $$\begin{array}{c} x=\sum_{k=0}^{N-1}\mu_{k}{\mathrm{PC}}_{k}=\sum_{k=0}^{L-1}\mu_{k}{\mathrm{PC}}_{k}+\sum_{k=L}^{N-1}\mu_{k}{\mathrm{PC}}_{k} \end{array}$$

where the first L terms correspond to PCs contributed to 95% of the variances of the data. The rest of the terms (N−L) in total contributed to 5% of the variances which are often regarded as noises. This allows us to simulate inherent noises by manipulating the weights of these “noisy” PCs, that is,

(8) $$\begin{array}{c} \tilde{x}=\sum_{k=0}^{L-1}\mu_{k}{\mathrm{PC}}_{k}+\sum_{k=L}^{N-1}\rho ∙\mu_{k}{\mathrm{PC}}_{k} \end{array}$$

where ρ>0 and $\tilde{x}$ is the noisy version of the original sample x. More details of generating PCA noises can be found in the support document.

## 3 Results

### 3.1 Database and Evaluation Protocol

The database used in our study is one of large publicly available database which is a collection of multiple human bulk RNA‐Seq datasets compiled by the reference (Shokhirev and Johnson 2021). It is publicly available in the Sequence Read Archive (https://www.ncbi.nlm.nih.gov/sra). The raw gene count table and metadata can be downloaded from Mendeley (https://doi.org/10.17632/92rgnswtn8.1).

This database consists of 31 datasets produced by different labs from the references, and contains 3060 samples in total which cover young, adult, and old human subjects with various health conditions such as Alzheimer's disease (AD), Schizophrenia, Age‐related Macular Degeneration (AMD), Dilated Cardiomyopathy (DCM), Dysplasia, and others. Samples were collected across different organs. Details can be found in Table 1. For data preprocessing and filtering, we followed the protocol in the reference (Shokhirev and Johnson 2021). For convenience, we will refer to this database as “MCATS” to highlight that it includes samples of multiple cohorts across tissues, healthy conditions and sex.

**TABLE 1:** Details of the samples used in this study including the organs where the samples were taken from, number of healthy/unhealthy samples from different organs, age distributions etc.

Organ	#References	Healthy	Unhealthy		Male	Female	Age (years)
Brain	3	91	Alzheimer's disease (AD)	133	144	80	32 ~ 103
			Schizophrenia	57	125	23	24 ~ 99
Retina	6	176	Age‐related Macular Degeneration (AMD)	406	285	297	47 ~ 107
Heart	2	162	Dilated Cardiomyopathy (DCM)	166	174	154	15 ~ 83
Lung	1	25	Dysplasia	50	51	24	47 ~ 77
Others	23	901	—	893	736	953	0.25 ~ 86
Total	35	1355	—	1705	1440	1515	0.25 ~ 107

*Note:* This database is a collection of 31 datasets from the references. Note that 105 samples of the category of “Others” do not have the gender specified.

To evaluate the performance of an aging clock, two cases need to be considered:

For healthy subjects, it is straightforward and we can simply use the mean‐absolute‐error between the predicted and chronological age, since the biological age of a healthy subject is (assumed to be) equal to its chronological age.

For unhealthy subjects, their biological age often does not match the chronological age, we adopt a qualitative analysis used in (Jonsson et al. 2019), termed as *PAD significance*, where PAD stands for “Predicted Age Difference” or more accurately the difference between the predicted and chronological age. PAD has been widely used for estimating the deviation of a subject from normal aging. Note that PAD on subjects of interest itself is insufficient to determine normal or abnormal aging, unless the aging clock works perfectly for healthy subjects with zero prediction error which rarely happens in reality. So instead of looking into PADs of subjects of interest themselves, one prepares a control group of healthy subjects unseen by the aging clock during training, and calculate their PADs. By testing whether these two groups of PADs are statistically different from one another, one can determine whether abnormal aging exists (Supporting Information: Section S1).

In our study, besides healthy subjects, we chose three unhealthy conditions that are known to be related to accelerated aging, namely Alzheimer's disease, Schizophrenia, age‐related macular degeneration (AMD), and two unhealthy conditions that are not known to be related to unhealthy aging, namely dilated cardiomyopathy (DCM) and dysplasia (Jonsson et al. 2019; Chang et al. 2019; Bashyam et al. 2020). Details of samples of these conditions including gender, age distribution are shown in Table 1.

### 3.2 Competing Methods and Implementation Details

Almost all non‐deep‐learning methods require explicit gene selection, we therefore adopted four popular methods, namely Gene expression filtering (Fleischer et al. 2018), variable and differential genes (Shokhirev and Johnson 2021), and AgingMap (Mao et al. 2023). For iterative searching of genes (Meyer and Schumacher 2021), we had to exclude it from comparison because it demands too much computational resources. With our hardware and experimental settings, it takes months to complete a single run. If we adopt the leave‐one‐out scheme as in (Meyer and Schumacher 2021), it would take many years to finish the experiments.

Methods of LDA, random forest and elastic net were implemented based on sklearn (Pedregosa et al. 2011). Our investigation showed that only random forest among these conventional methods benefited from data augmentation with Gaussian noise, and the corresponding results were obtained with data augmentation. CNN‐2D were proposed with data augmentation and our implementation followed the design and training protocol in (Mohamadi et al. 2021).

DeepQA was implemented in Python (version 3.8.10) and based on the popular deep learning framework PyTorch (Paszke et al. 2019) (version 1.12.1). The number of experts in MoE was 2. The optimizer was Adam (Kingma and Ba 2015). The learning rate was set initially as 5e‐3 and decayed in step wise by 0.8. The batch size was 512. For each training sample, 30 artificial samples augmented with Gaussian noises were generated for training. The experiments were conducted on servers with multiple NVIDIA GeForce RTX 3090 and 3080 GPUs.

### 3.3 Accuracy in Biological Age Prediction

As discussed previously, the evaluation protocols of “training and test” in many existing works are flawed when gene selection is involved and lead to selection bias. So in our study, following the suggestion in (Ambroise and McLachlan 2002), we carried out 10‐fold cross‐validation. For the implementation of competing methods, we have ensured no “*test set leakage*” would occur by performing gene selection within each fold. We reported the averaged prediction accuracy.

Table 2 presents the comparison of the proposed DeepQA with existing state‐of‐the‐art methods on both healthy and unhealthy subjects. The first two row shows the prediction performance of models trained on healthy subjects of each cohort, respectively. For each cohort, we trained a model to predict their biological age. This is a common practice for biological age prediction in existing work. Row 3 ~ 10 show results of models trained on healthy subjects of multiple cohorts, and the last row shows the performance of our DeepQA trained on both healthy and unhealthy subjects from multiple cohorts. A graphical comparison of DeepQA and the competing methods can also be found in Figure 3a.

**TABLE 2:** Prediction accuracy of the compared aging prediction methods on healthy and unhealthy subjects.

Method	Trained on	Healthy		Unhealthy					
		MAE↓	*R* ^2^↑	AD	AMD	Schi.	DCM	Dysp.	Score
				(A.)	(A.)	(A.)	(N.)	(N.)	(5)
RF‐Differential	Healthy, per cohort	8.307	0.574	N.	A.	A.	N.	N.	4
RF‐Variable	Healthy, per cohort	7.967	0.599	N.	A.	A.	N.	N.	4
ELDA‐GEF	Healthy	6.366	0.689	N.	N.	A.	A.	A.	1
CNN‐2D	Healthy	8.778	0.488	A.	A.	A.	A.	N.	4
RF‐Differential	Healthy	7.284	0.686	N.	N.	A.	A.	A.	1
RF‐Variable	Healthy	6.821	0.711	N.	N.	A.	A.	A.	1
EN‐Differential	Healthy	8.512	0.580	A.	N.	A.	A.	A.	2
EN‐Variable	Healthy	7.995	0.634	N.	N.	A.	A.	A.	1
EN‐AgingMap	Healthy	8.292	0.582	N.	N.	A.	A.	A.	1
EN‐All	Healthy	6.931	0.693	N.	N.	A.	A.	A.	1
**DeepQA(ours)**	Healthy	5.295	0.744	A.	N.	A.	A.	A.	2
	**Healthy + unhealthy**	**4.820**	**0.789**	**A**.	**A**.	**A**.	**N**.	**N**.	**5**

*Note:* For unhealthy subjects of five conditions, A. stands for accelerated aging and N. stands for no accelerated aging. Schi. stands for Schizophrenia. Dysp. stands for Dysplasia.

For healthy subjects, DeepQA outperformed all the compared methods significantly in terms of both MAE and $R^{2}$ and achieved the lowest prediction error of 4.820 in MAE which is 1.5 year more accurate than the second best method *ELDA‐GEF*. For unhealthy subjects, only our DeepQA correctly identified the aging status of all the conditions. Please refer to Tables S1–S11 for detailed results on unhealthy subjects. It is worth noting that even for DeepQA itself, training on both healthy and unhealthy subjects improved the prediction performance significantly, demonstrating the necessity of exploiting data of unhealthy subjects (Table 2).

The good performance of ELDA‐GEF on healthy subjects was mainly due to an ensemble of a substantial number of trained models. However, this design also makes this method very inefficient. Using ensemble technique here indeed improved the prediction performance, but also increased the risk of overfitting. Another interesting observation is that elastic net performed the best when fed with all the genes which indicated that its sparsity constraint played a key role in selecting genes in the process of model fitting. On the other hand, as we will discuss in the next subsection, the robustness against noise of these methods was unsatisfactory which indicated a certain degree of overfitting.

Interestingly, the accuracy of the CNN‐2D method on healthy subjects is rather low, while it did perform well on unhealthy subjects. This may indicate that deep neural networks could be especially suitable for the task of aging estimation, yet the specific architecture of 2D convolutional networks may not be a good choice though. This is due to the inherent flaw of converting gene expression data into 2D images, as neighborhood relations of genes can be arbitrary. In other words, unlike real images, these pseudo‐images of gene expression data contain false neighboring information which leads to poor performance, especially when combined with the age‐bin strategy.

**FIGURE 2:**
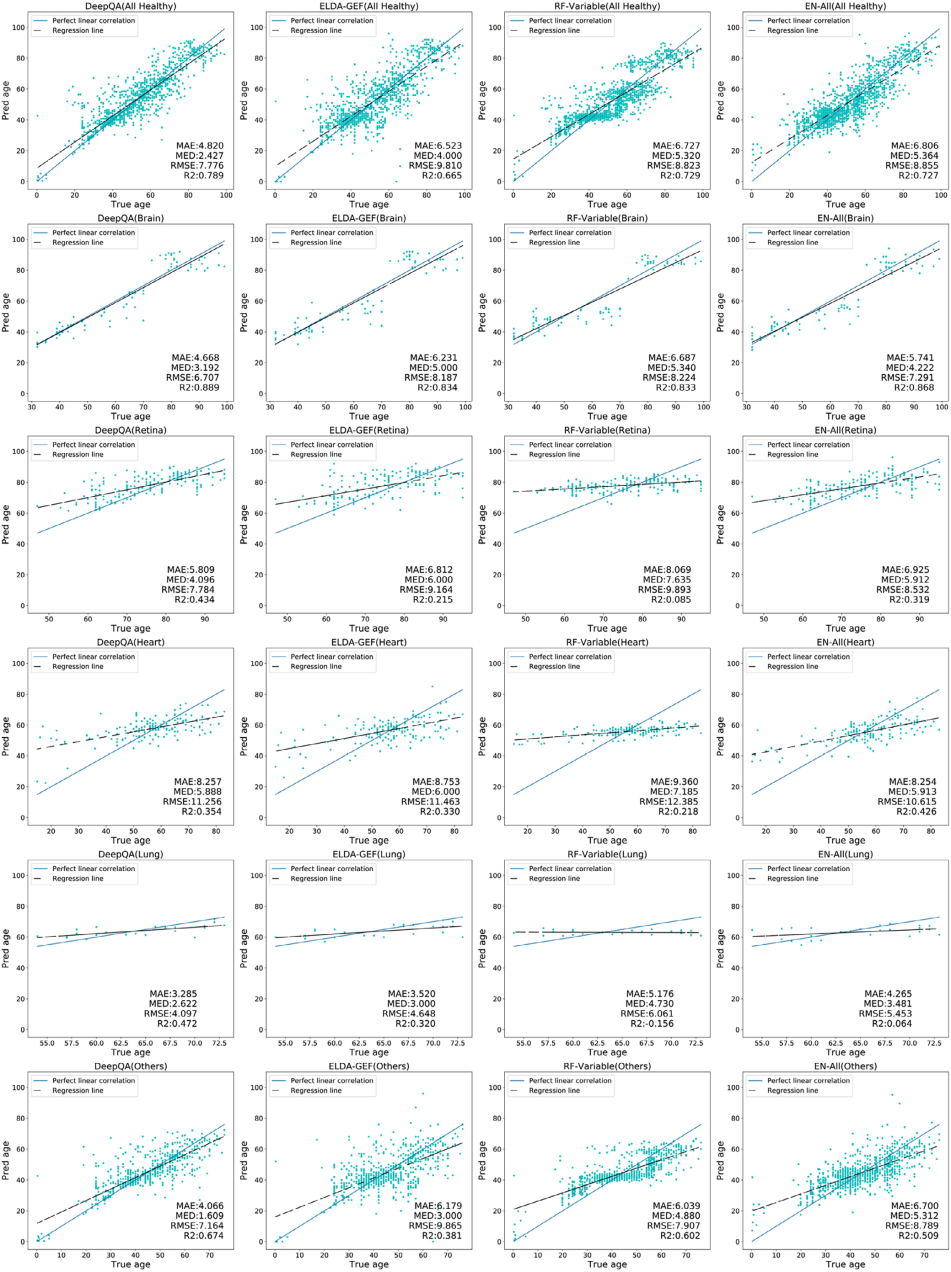
Prediction performance of DeepQA compared with existing methods on healthy subjects of different groups, namely all, brain, retina, heart, lung, and others from top to bottom. Columns correspond to DeepQA, ELDA‐GEF, RF‐Variable, and EN‐All, respectively. In each plot, the x‐axis is the true age and the y‐axis is the predicted age. DeepQA outperformed existing methods on all groups.

**FIGURE 3:**
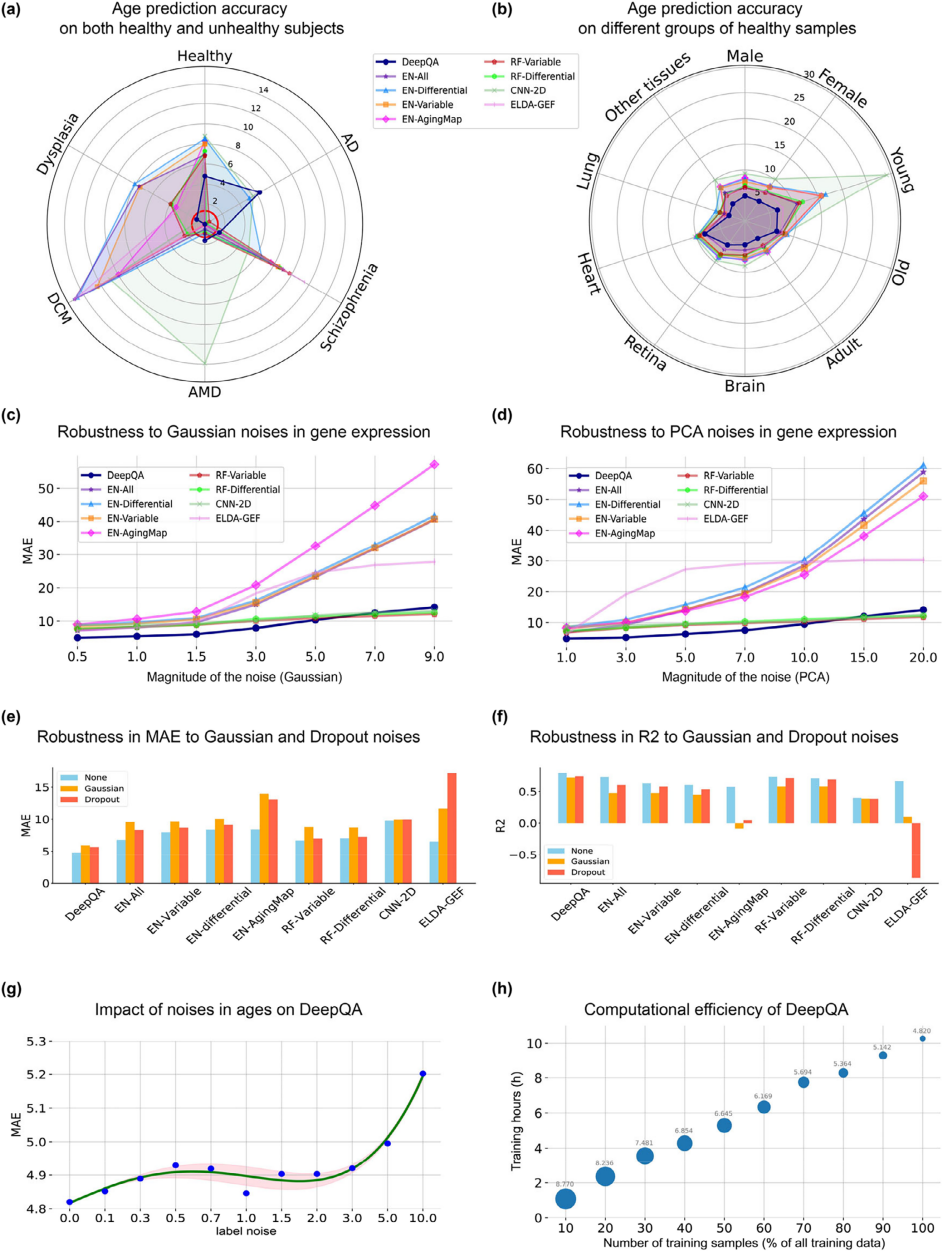
(a) Age prediction accuracy of DeepQA and existing methods on both healthy and unhealthy subjects. This plot is a graphical illustration of Table 2. For healthy subjects, MAE was used to measure the goodness of prediction. For unhealthy subjects, statistical significance, −log10(*p*‐value), was plotted to show the plausibility of prediction. (b) Comparison of DeepQA and existing methods on different groups of healthy samples. DeepQA outperformed existing methods in all the groups. (c) Robustness of DeepQA and other methods against simulated Gaussian noises in gene expression data at different levels. (d) Robustness of DeepQA and other methods against simulated PCA noises in gene expression data at different levels. (e) Robustness in MAE of DeepQA and other methods against Gaussian or Dropout noises at a typical magnitude of 1.5. (f) Robustness in *R* ^2^ of DeepQA and other methods against Gaussian or Dropout noises at a typical magnitude of 1.5. (g) Impact of noises in ages (labels) on prediction accuracy of DeepQA on healthy subjects. (h) Training time of DeepQA w.r.t the size of the training data. The diameters of the blue solid dots correspond to the prediction error in MAE.

Since our database is heterogeneous and contains multiple datasets or cohorts, we therefore also calculated the prediction performance of DeepQA along with existing competing methods on healthy subjects from different groups of samples, namely male, female, young, adult, old, brain, retina, heart, lung, and other diseases in Figures 2 and 3b. It is evident that the proposed DeepQA outperformed all the other methods. This suggested that DeepQA has the potential to be a universal aging clock which learns to predict biological age of samples from transcriptome data across different organs, sex and age with sufficient data for training.

### 3.4 Robustness to Noises in Gene Expression

The raw data of RNA sequencing are often contaminated by multiple types of noises, for example, technical noise, biological variation, experimental settings, and many other factors (Conesa et al. 2016). Though that denoising and imputation methods for data cleaning are available, the performance of which is not always satisfactory (Eraslan et al. 2019; Moutsopoulos et al. 2021; Li, Brouwer, and Luo 2022). As a result, the downstream analysis such as age prediction here becomes even more challenging due to the presence of noises. It is therefore important to evaluate and improve the robustness of the prediction methods against noises.

Following the general practice in both biology and machine learning (Bishop 2006; Li, Brouwer, and Luo 2022), and more importantly to simulate the effect of many different noise factors combining together (according to the Central Limit Theorem), we injected Gaussian noise into the gene expression data, and tested the robustness of the proposed method accordingly at different levels of noises (Figure 3c). When the magnitude of noises was smaller than 5, our method significantly outperformed other competing methods. While extremely large noises were imposed and too much of information and structure in data were destroyed, none of these methods were able to predict with an error of less than 10. Some of them performed extremely bad though.

To have a clear view of the comparison, we have also plotted the prediction accuracies of the competing methods at a typical level of noises (1.5 for Gaussian, 3% for Dropout) (Figure 3e,f). DeepQA is much more robust than any other competing methods with a large margin (> 3 years in MAE). In contrast, the second best method ELDA‐GEF performed extremely unsatisfactorily against the interference of the noises, which indicated that overfitting might have occurred in its good result in Table 2 (Bishop 2006; Xu, Caramanis, and Mannor 2009; Goodfellow, Bengio, and Courville 2016). Similarly, EN with all genes performed much better than EN with variable or differential genes, but showed comparable robustness which also indicated a certain degree of overfitting. It is expectable that CNN‐2D has shown to be quite robust, but its prediction accuracy was too low to be an appealing prediction model. Besides Gaussian noise, we have also tested robustness of these methods against dropout noises (Figure 3e,f).

Besides injecting noises directly into the gene expression data, we are also interested in how DeepQA and other methods would respond to the inherent noises in the gene expression data. Hence, following the popular approach of using PCA to isolate small variations which may be regarded as “noises.” Results are reported in Figure 3d. Overall, our DeepQA showed superior robustness among the compared methods. Interestingly, the compared methods exhibited three distinct dynamics: DeepQA, CNN‐2D, RF‐Variable and RF‐Differential formed a leading group which significantly outperformed the other two groups. When the magnitude of the noises reached to 20, the MAEs were still about impressively 10. The second group of methods were elastic‐net‐based ones where the prediction errors almost linearly grew when the noises become larger, indicating a very poor robustness. Lastly, the response curve of ELDA‐GEF is radically different from the other two groups where it grew very fast when the noise was small, but saturated beyond 7. These behaviors may indicate that the second and three groups of methods were biased to rely more heavily on factors with small variations (including inherent noises) or large. In contrast, the first group of methods including DeepQA may be able to learn features in a balanced way.

### 3.5 Robustness to Noises in Age Labels

The MoE model in DeepQA are trained with pairs of data<gene expression data, age label>and we have shown that our method was remarkably robust to the noises in the input gene expression data. Moreover, the age labels may also be corrupted by noises. Particularly they in fact already contain a certain degree of inherent uncertainty due to the inaccuracy in quantifying age in years. A simple and straightforward example is that the “true” chronological age of two persons with the same age label (e.g., 60 years) could differ in 1 year if they were born in the beginning and the end of the same year, respectively.

To examine how our method responds to this kind of “label noise,” we designed and conducted a set of experiments where Gaussian noises were added on age labels of all training samples. Results were reported in Figure 3g. The x‐axis represents the std. of the Gaussian noises, and the y‐axis shows the prediction accuracy on healthy subjects. Overall, DeepQA was able to handle label noises of reasonably large magnitude very well (std < 3 years). When label noises were too strong, it is no surprise that the performance of DeepQA decreased.

### 3.6 Computational Efficiency

We examined the computational efficiency and scalability of DeepQA and reported the training hours and prediction accuracy on a single NVIDIA GeForce RTX 3090 GPU when different number of training samples were used (Figure 3h). The training time grew basically linearly when the size of the training data increased. Note that we haven't used sophisticated techniques to speed up the training process, other than standard optimization. When a large dataset is available, techniques such as distributed and parallel training can significantly boost the training speed (Li et al. 2020).

### 3.7 Impact of the Hyperparameters

**FIGURE 4:**
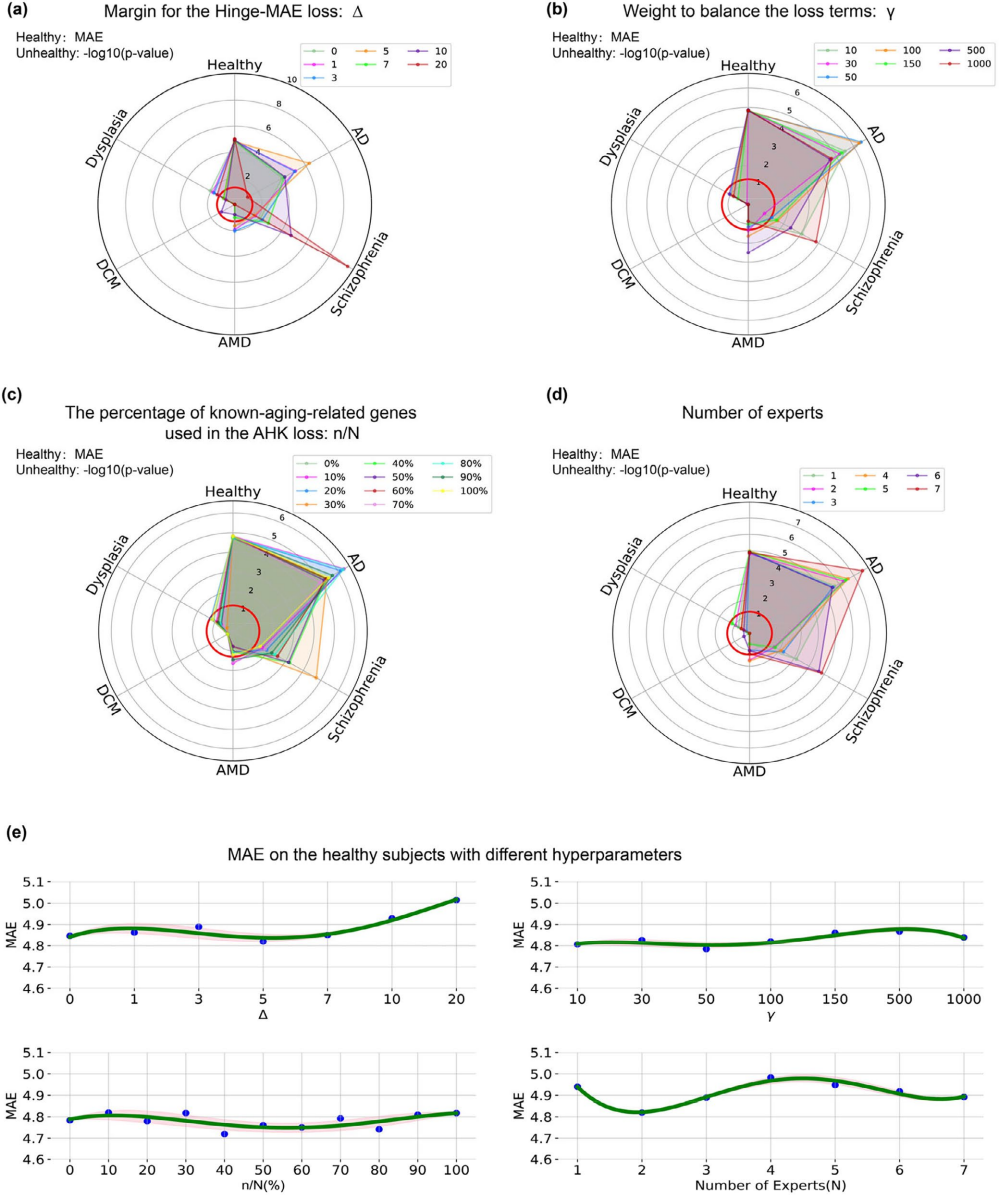
Age prediction accuracy of DeepQA with different hyperparameter. (a) ∆, the margin for the Hinge‐MAE loss. (b) γ, weight to balance the loss terms. (c) $\frac{n}{N}$, the percentage of genes used in the AHK loss. (d) The number of experts. The red circle corresponds to ‐log10(0.05) which is the threshold for the significance test. For AD, AMD, and Schizophrenia, the data points should be located outside the red circle which indicates aging acceleration has been detected. For the other two conditions, data points should be inside the red circle. (e) MAE on the healthy subjects with different hyperparameters, among which the number of experts was the most sensitive one affecting the prediction accuracy on the healthy subjects. The optimal values of these hyperparameters were shown in the main text.

The values of hyperparameters in DeepQA were determined manually following the standard protocol (Goodfellow, Bengio, and Courville 2016). To investigate how DeepQA is affected by its key hyperparameters, we conducted a series of experiments where DeepQA was trained with different values of hyperparameters and the corresponding prediction accuracy on both healthy and unhealthy subjects were reported (Figure 4). Namely, these hyperparameters were the margin for the Hinge‐MAE loss ∆, the weight to balance the loss terms γ, the percentage of genes used in the AHK loss $\frac{n}{N}$, and the number of experts. For clarity, we have also plotted the MAE alone when different hyperparameters were chosen (Figure 4e).

The parameters $\frac{n}{N}$ and γ control how the AHK loss contributes to the learning process, and interestingly the prediction error in MAE on healthy subjects varied slightly when both of these two parameters became larger (Figure 4e), while the significances on unhealthy subjects showed radical changes (Figure 4b,c). This demonstrated that the AHK loss was crucial for detecting aging acceleration (or not).

In the Hinge‐MAE loss, when the parameter ∆ became larger than 7, the prediction error in MAE on healthy subjects increased significantly, and the significances on unhealthy subjects of AD, AMD and Dysplasia flipped wrongly (Figure 4a,e). The number of experts controls the model capacity of DeepQA, and its impact on age prediction accuracies were also quite typical. Small or large values of this parameter decreased the performance as the model was experiencing underfitting or overfitting (Figure 4d,e). In practice, if a large amount of data is available for training, we can increase the number of experts to enlarge the model to learn from big data.

Interestingly, our method is relatively insensitive to its hyperparameters. This suggests that the training of DeepQA converged to flat minima of the loss function (Equation 2), which usually indicated good generalization (Keskar et al. 2017; Cha et al. 2021).

### 3.8 Explainability and Clinical Applicability via Identifying Important Genes

**FIGURE 5:**
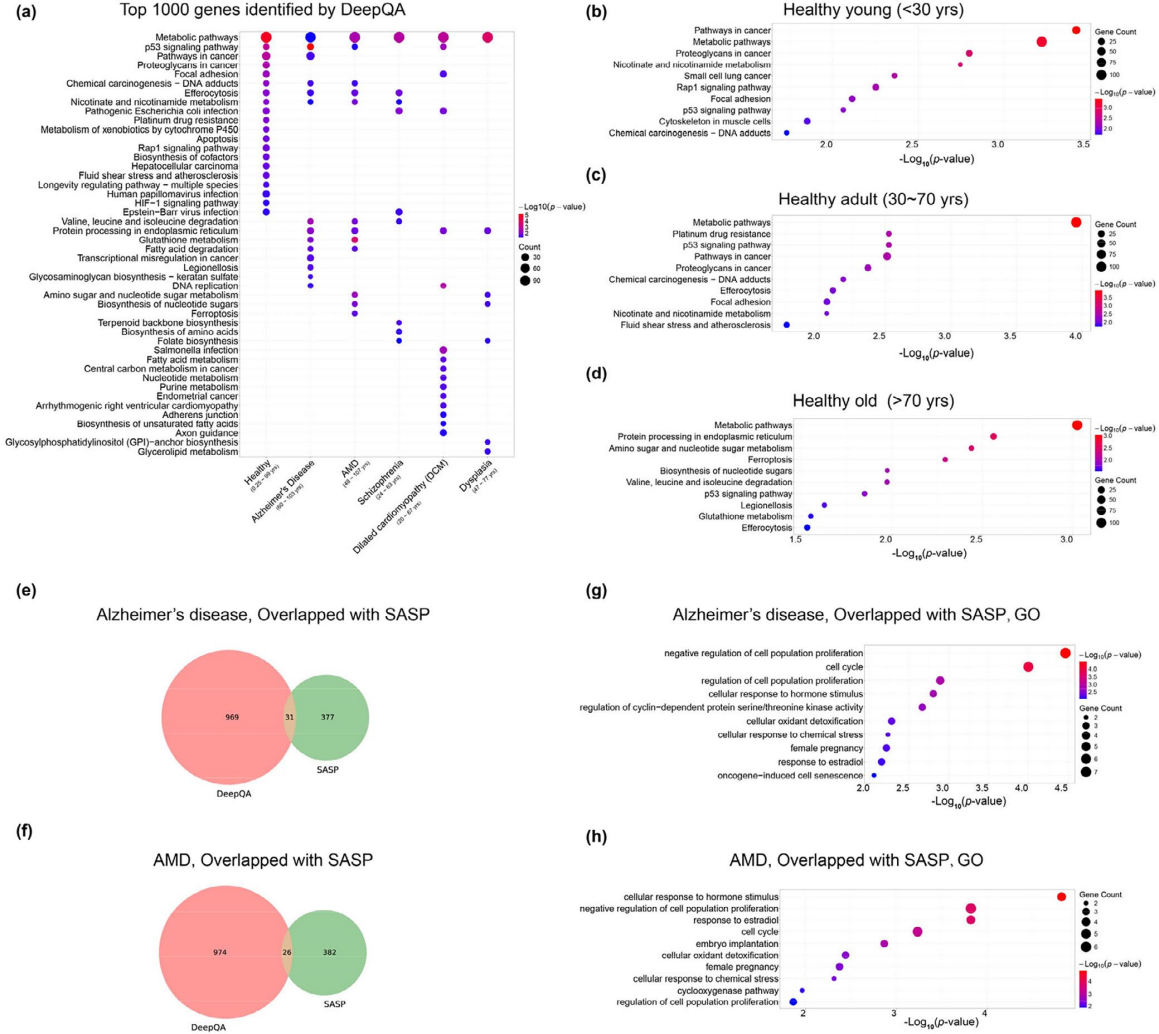
(a) KEGG pathways enriched on the top‐1000 genes identified to be important for age prediction on the group of healthy subjects and five unhealthy conditions by DeepQA. (b–d) KEGG analysis of genes identified by DeepQA from young (b), adult (c), and old healthy groups (d), respectively. (e–f) DeepQA genes overlapped with SASP genes in AD (e) or AMD (f). (g–h) GO analysis of overlapped genes from AD (g) or AMD (h). In all graphs, top 1000 genes with DeepQA were selected. In all the bubble charts, color gradient indicates the level of statistical significance (−log10 (p‐value)) and the dot size indicates the number of genes. The gene enrichment analysis was performed using the online DAVID tools (Sherman et al. 2022).

In general, understanding how deep neural networks work is very challenging. For aging clocks, we can start from investigating important genes identified by DeepQA and checking their biological plausibility. To this end, we performed the gene enrichment analysis on the top‐1000 important genes identified by DeepQA on all the samples (Figure 5a). We showed results of healthy subjects, Alzheimer's disease, Schizophrenia, age‐related macular degeneration, dilated cardiomyopathy and dysplasia.

The healthy samples (Figure 5a) can be categorized into three groups, namely young (< 30 years), adult (30 ~ 70 years), and old (> 70 years), so we also used DeepQA to identify genes prominent in each of these three groups, and performed KEGG, respectively. The results indicate that in the young group, there is an enrichment of pathways in cancer, metabolic pathways, and nicotinate and nicotinamide metabolism (Figure 5b). The enrichment of various cancer pathways indicated stem cells properties (Reya et al. 2011). In the adult group, the enrichment is observed in metabolic pathways, pathways in cancer, and the cell cycle pathway related to p53 (Figure 5c) (Hafner et al. 2019). Metabolic processes, ferroptosis, legionellosis (common in the elderly people [Flanagan 2020]), and protein degradation pathways such as protein processing in endoplasmic reticulum are enriched in the old group (Figure 5d) (Krshnan, van de Weijer, and Carvalho 2022).

We also examined the senescence‐associated secretory phenotype (SASP)‐related genes among the top 1000 genes identified by DeepQA on healthy and unhealthy samples (Figure 5e,f; Figure S2; Table S12) (Suryadevara et al. 2024; Qi et al. 2024), followed by GO analysis detecting gene functions of the overlapped genes. While subjects of AD and AMD share similar age range, AD was enriched of negative regulation of cell population proliferation, cell cycle, and regulation of cell population proliferation (Figure 5g). While AMD was enriched of cellular response to hormone stimulus, negative regulation of cell population proliferation, response to estradiol and cell cycle (Figure 5h). These showed that both aging‐related diseases including AD and AMD are enriched in cell cycle and cell proliferation.

DeepQA was able to learn to identify and made use of genes for age prediction in a biological plausible manner. It learned to predict biological ages using not only genes that are associated to aging in general, but also ones that are linked to the characteristics/conditions of a particular group that a sample belongs to. This serves as a biological validation of the explainability of DeepQA.

More importantly, the ability of DeepQA identifying important genes can also be an effective tool for discovery of novel gene biomarkers, alternative to (not necessarily replacing) such as differentially expressed genes (DEGs) analysis. This is another clinical usage of DeepQA besides age prediction as a diagnosis or evaluation tool for certain diseases. Here we demonstrated how DeepQA can be jointly used with DEG analysis to identify potential important genes.

**FIGURE 6:**
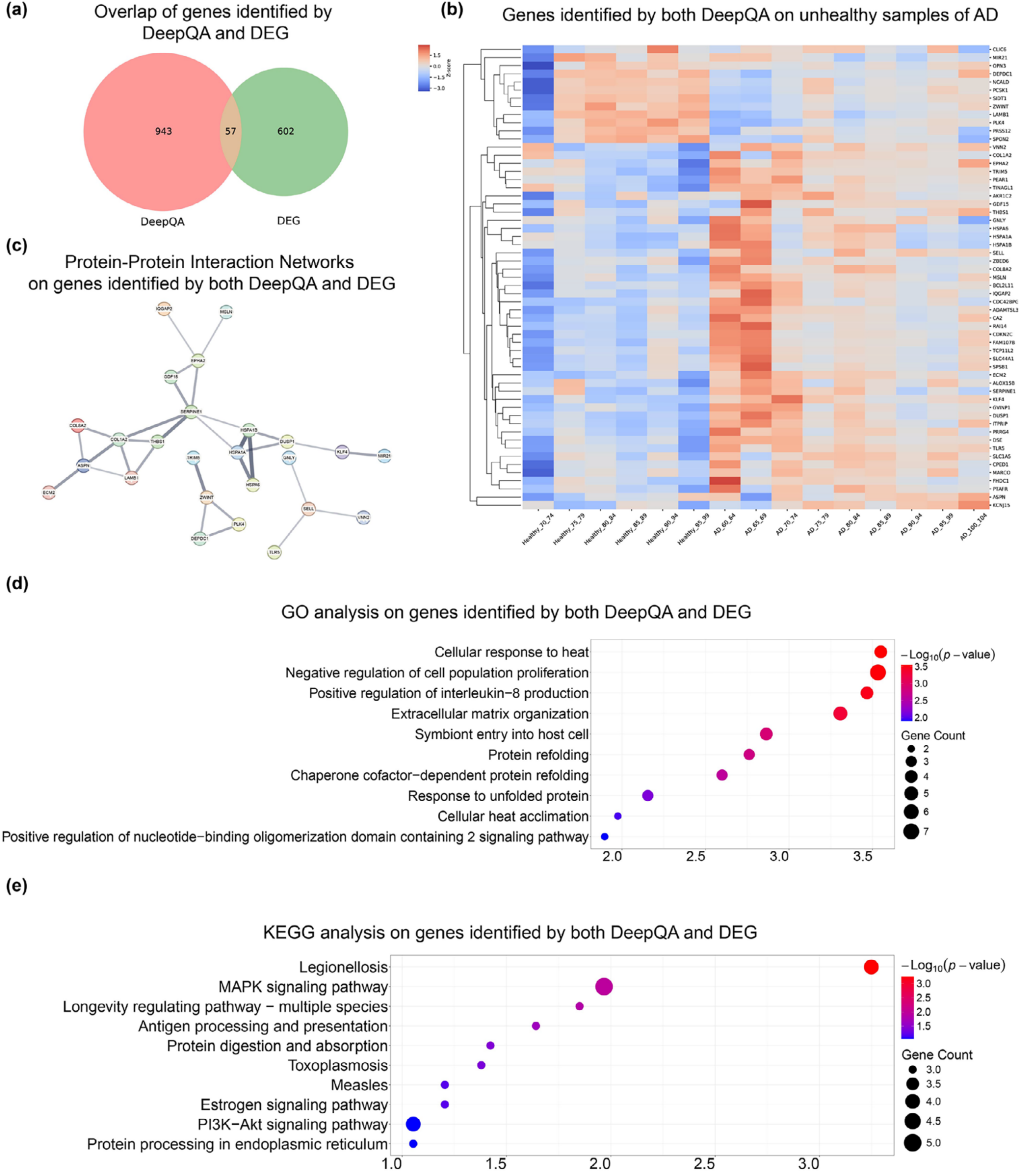
DeepQA and DESeq2 joint analysis of RNA‐seq dates from Alzheimer's disease on samples from (Friedman et al. 2018). (a) Overlap between DEGs of AD and top 1000 genes of DeepQA from AD. (b) Expression heatmap of overlapped genes. Average expression level of each age bin was determined and presented as Z‐score across different bins. (c) The protein–protein interaction (PPI) network of the overlapped genes. GO analysis (d) or KEGG analysis of pathways (e) related to upregulated genes of DEGs from overlapped genes. The analysis was done with DAVID. Color shade indicated significance and dot size indicated gene count of the corresponding pathway.

We chose a batch of brain samples containing both healthy controls and unhealthy samples with AD (Friedman et al. 2018). We used both DESeq2 and DeepQA to analyze the samples of AD or healthy. The top 1000 genes of DeepQA were overlapped with the DEGs, showing that 57 genes were overlapped (Figure 6a). Among overlapped genes, most of them were upregulated DEGs in AD (Figure 6b). GO analysis revealed biological processes related to negative regulation of cell population proliferation, positive regulation of interleukin−8 production and extracellular matrix organization (Figure 6d). KEGG analysis of overlapped target genes identified upregulation of various aging related pathways, including MAPK signaling pathway, longevity regulating pathway and PI3K − Akt signaling pathway (Figure 6e) (López‐Otín et al. 2023; Nikoletopoulou, Kyriakakis, and Tavernarakis 2014). In addition, PPI network for overlapped genes was generated using an online tool String (Szklarczyk et al. 2023) (Figure 6c). Nodes with different colors represented different proteins and the edges between nodes represented known interactions or predicted interactions between these proteins. In summary, these results showed that DeepQA can produce meaningful genes overlapped with DEGs which are associated with aging in AD. In other words, it can help to further filter out genes generated by DEG analysis, and be an effective and efficient tool in complementary to DEG analysis. Please note that under this scenario, train‐test split is no longer a concern, and any sample can be used to produce potential genes of importance.

## 4 Discussion

We proposed a deep‐learning‐based aging clock, named as DeepQA, which predicts biological age of an individual based on transcriptome data, and discovers genes of importance via analyzing saliency maps efficiently. With a simple yet effective Hinge‐MAE loss and the architecture of mixture of experts which is especially suitable for modeling heterogeneous data, DeepQA can train on both healthy and unhealthy subjects of multiple cohorts to improve the performance of estimating biological age, especially for unhealthy subjects. Compared to existing methods, our method enjoys the following advantages.

Firstly, it can predict biological age of healthy and unhealthy subjects from gene expression data much more accurately than existing methods. In our experiments, DeepQA outperformed existing methods with a large margin on a database which consists of several public datasets. On healthy subjects, our DeepQA achieved superior prediction performance which was 1.5 years better than the second best method. For unhealthy subjects, DeepQA was the only one with correct prediction on subjects of all five unhealthy conditions.

Secondly, it does not require gene selection as a preprocessing step and can identify important genes via analyzing saliency maps after model fitting. DeepQA avoids the extremely inefficient exhaustive search of genes, and is therefore also immune to the selection bias in gene selection process which numerous existing methods have suffered from. It provides a novel means to identify genes activated in aging prediction, alternative to such as differential gene expression analysis. We validated this by showing important genes selected by DeepQA were indeed meaningful, and consistent with known human knowledge.

It should be emphasized that all existing methods (aging clocks) of estimating biological age were proposed to train on (data of) healthy subjects only. To the best of our knowledge, our DeepQA is the first method which can train on both healthy and unhealthy subjects and shows superior performance of predicting biological age compared to existing methods. The key is a new loss we proposed, called Hinge‐MAE loss, combined with the network architecture of mixture of experts, to train the AI model on unhealthy subjects. This loss for unhealthy subjects is general and can be used for any AI model.

One of key findings in our study is that existing methods performed rather poorly on unhealthy subjects when trained on heterogeneous samples of multiple cohorts, compared to on each homogeneous cohort, as shown in Table 2. A disadvantage of the latter is multiple models are required to be trained and deployed. This is certainly an inefficient solution which also cannot benefit from large (heterogeneous) data. In contrast, our DeepQA demonstrated clearly its ability and advantages of learning from heterogeneous data, thanks to the architecture of mixture‐of‐experts and the Hinge‐MAE loss. In this sense, DeepQA provides a “unified” solution for estimating biological age from gene expression data.

To demonstrate the clinical usage of DeepQA, we examined the SASP genes predicted in six groups and found that they were enriched in metabolism, cell cycle, and cellular proliferation, showing similarities in physiological processes. Moreover, among healthy individuals of different ages, the pathways in the old group significantly differed from those in the other two groups, while the data range for AD and AMD spanned from adult to old age, sharing similar enriched pathways with the healthy old group, indicating the plausibility of the prediction results. For non‐aging‐related diseases, such as DCM, enriched pathways were mostly related to the characteristics of the disease itself, instead of age, including fatty acids, purine, and nucleotide (Gigli et al. 2024). In subsequent analyses using a batch of AD data, DeepQA was jointly used with DESeq2. Compared with healthy individuals, there were 57 age‐related genes predicted by DeepQA that showed differential expression in AD. Among these, MAPK and PI3K‐Akt are related to the pathogenesis of AD or aging (Kitagishi et al. 2014; Dai et al. 2016), while legionellosis and longevity regulating pathways exhibit characteristics of the elderly. Notably, the impact of legionellosis and measles on the aging process in AD has not been reported and merits further exploration.

Our current aging clock is gene‐centric, focusing on genes within the transcriptome. For future work, we will investigate the possibility of including more data, for example, transcripts from noncoding sequences like transposable elements which contribute to the aging process and can serve as aging markers (Lu 2023; Bourque et al. 2018; Liu et al. 2023; De Cecco et al. 2019), and for example, DNA methylation data, to form multimodal aging clocks to further improve the prediction accuracy through multimodal learning. Moreover, we shall also further investigate the DeepQA's explainability and how it can be used to discover novel biomarkers, besides predicting biological age.

## Author Contributions

**Hongqian Qi:** conceptualization, methodology, data curation and analysis, writing – original draft, writing – review and editing. **Hongchen Zhao:** methodology, data curation and analysis, writing – review and editing. **Enyi Li:** methodology, data curation and analysis, writing – review and editing. **Xinyi Lu:** data analysis, funding acquisition, writing – review and editing. **Ningbo Yu:** conceptualization, data analysis, funding acquisition, writing – review and editing. **Jinchao Liu:** conceptualization, data analysis, funding acquisition, supervision, writing – original draft, and writing – review and editing. **Jianda Han:** conceptualization, data analysis, funding acquisition, supervision and writing – review and editing.

## Conflicts of Interest

The authors declare no conflicts of interest.

## Supporting information


Data S1.

Table S12.

## Data Availability

The data that we have used in this study are publicly available. The details can be found in the main text. The code for DeepQA will be released publicly at https://github.com/Chaoscendence/DeepQA.

## References

[acel14471-bib-0001] Agarwal, R. , L. Melnick , N. Frosst , et al. 2021. “Neural Additive Models: Interpretable Machine Learning With Neural Nets.” Advances in Neural Information Processing Systems 34: 4699–4711.

[acel14471-bib-0002] Aging Biomarker Consortium , Y. Jia , J. Wang , et al. 2023. “A Framework of Biomarkers for Brain Aging: A Consensus Statement by the Aging Biomarker Consortium.” Lifestyle Medicine 2, no. 3: lnad017. 10.1093/lifemedi/lnad017 PMC11749242 39872296

[acel14471-bib-0003] Ambroise, C. , and G. J. McLachlan . 2002. “Selection Bias in Gene Extraction on the Basis of Microarray Gene‐Expression Data.” Proceedings of the National Academy of Sciences of the United States of America 99, no. 10: 6562–6566. 11983868 10.1073/pnas.102102699 PMC124442

[acel14471-bib-0004] Bao, H. , J. Cao , M. Chen , et al. 2023. “Biomarkers of Aging.” Science China Life Sciences 66, no. 5: 893–1066. 37076725 10.1007/s11427-023-2305-0 PMC10115486

[acel14471-bib-0005] Bashyam, V. , G. Erus , J. Doshi , et al. 2020. “MRI Signatures of Brain Age and Disease Over the Lifespan Based on a Deep Brain Network and 14 468 Individuals Worldwide.” Brain: A Journal of Neurology 143, no. 7: 2312–2324. 32591831 10.1093/brain/awaa160 PMC7364766

[acel14471-bib-0006] Bishop, C. 2006. Pattern Recognition and Machine Learning. Berlin, Germany: Springer.

[acel14471-bib-0007] Bocklandt, S. , W. Lin , M. E. Sehl , et al. 2011. “Epigenetic Predictor of Age.” PLoS One 6, no. 6: e14821. 21731603 10.1371/journal.pone.0014821 PMC3120753

[acel14471-bib-0008] Bourque, G. , K. H. Burns , M. Gehring , et al. 2018. “Ten Things You Should Know About Transposable Elements.” Genome Biology 19: 1–12. 30454069 10.1186/s13059-018-1577-z PMC6240941

[acel14471-bib-0009] Ceglia, N. , Z. Sethna , S. S. Freeman , et al. 2023. “Identification of Transcriptional Programs Using Dense Vector Representations Defined by Mutual Information With GeneVector.” Nature Communications 14, no. 1: 4400. 10.1038/s41467-023-39985-2 PMC10359421 37474509

[acel14471-bib-0010] Cha, J. , S. Chun , K. Lee , et al. 2021. “SWAD: Domain Generalization by Seeking Flat Minima.” Advances in Neural Information Processing Systems 34: 22405–22418.

[acel14471-bib-0011] Chang, A. Y. , V. F. Skirbekk , S. Tyrovolas , N. J. Kassebaum , and J. L. Dieleman . 2019. “Measuring Population Ageing: An Analysis of the Global Burden of Disease Study 2017.” Lancet Public Health 4, no. 3: e159–e167. 30851869 10.1016/S2468-2667(19)30019-2 PMC6472541

[acel14471-bib-0012] Chen, Z. , Y. Deng , Y. Wu , Q. Gu , and Y. Li . 2022. “Towards Understanding the Mixture‐Of‐Experts Layer in Deep Learning.” Advances in Neural Information Processing Systems 35: 23049–23062.

[acel14471-bib-0013] Cheng, Y. , R. Hu , H. Ying , X. Shi , J. Wu , and W. Lin . 2024. “Arithmetic Feature Interaction Is Necessary for Deep Tabular Learning.” Proceedings of the AAAI Conference on Artificial Intelligence 38: 11516–11524.

[acel14471-bib-0014] Conesa, A. , P. Madrigal , S. Tarazona , et al. 2016. “A Survey of Best Practices for RNA‐Seq Data Analysis.” Genome Biology 17, no. 1: 13. 26813401 10.1186/s13059-016-0881-8 PMC4728800

[acel14471-bib-0015] Dai, H. , W. Hu , L. Jiang , L. Li , X. Gaung , and Z. Xiao . 2016. “p38 MAPK Inhibition Improves Synaptic Plasticity and Memory in Angiotensin II‐Dependent Hypertensive Mice.” Scientific Reports 6, no. 1: 27600. 27283322 10.1038/srep27600 PMC4901328

[acel14471-bib-0016] De Cecco, M. , T. Ito , A. P. Petrashen , et al. 2019. “L1 Drives IFN in Senescent Cells and Promotes Age‐Associated Inflammation.” Nature 566, no. 7742: 73–78. 30728521 10.1038/s41586-018-0784-9 PMC6519963

[acel14471-bib-0017] Du, J. , P. Jia , Y. Dai , C. Tao , Z. Zhao , and D. Zhi . 2019. “Gene2vec: Distributed Representation of Genes Based on Co‐Expression.” BMC Genomics 20, no. 1: 7–15. 30712510 10.1186/s12864-018-5370-x PMC6360648

[acel14471-bib-0018] Elmarakeby, H. A. , J. Hwang , R. Arafeh , et al. 2021. “Biologically Informed Deep Neural Network for Prostate Cancer Discovery.” Nature 598, no. 7880: 348–352. 34552244 10.1038/s41586-021-03922-4 PMC8514339

[acel14471-bib-0019] Eraslan, G. , L. M. Simon , M. Mircea , N. S. Mueller , and F. J. Theis . 2019. “Single‐Cell RNA‐Seq Denoising Using a Deep Count Autoencoder.” Nature Communications 10, no. 1: 390. 10.1038/s41467-018-07931-2 PMC6344535 30674886

[acel14471-bib-0020] Flanagan, N. M. 2020. “Legionellosis in Long‐Term Care: An Update.” Caring Ages 21, no. 1: 3.

[acel14471-bib-0021] Fleischer, J. G. , R. Schulte , H. H. Tsai , et al. 2018. “Predicting Age From the Transcriptome of Human Dermal Fibroblasts.” Genome Biology 19: 1–8. 30567591 10.1186/s13059-018-1599-6 PMC6300908

[acel14471-bib-0022] Fortelny, N. , and C. Bock . 2020. “Knowledge‐Primed Neural Networks Enable Biologically Interpretable Deep Learning on Single‐Cell Sequencing Data.” Genome Biology 21, no. 1: 190. 32746932 10.1186/s13059-020-02100-5 PMC7397672

[acel14471-bib-0023] Friedman, B. A. , K. Srinivasan , G. Ayalon , et al. 2018. “Diverse Brain Myeloid Expression Profiles Reveal Distinct Microglial Activation States and Aspects of Alzheimer's Disease Not Evident in Mouse Models.” Cell Reports 22, no. 3: 832–847. 29346778 10.1016/j.celrep.2017.12.066

[acel14471-bib-0024] Gigli, M. , D. Stolfo , M. Merlo , G. Sinagra , M. R. Taylor , and L. Mestroni . 2024. “Pathophysiology of Dilated Cardiomyopathy: From Mechanisms to Precision Medicine.” Nature Reviews Cardiology: 1–16. 10.1038/s41569-024-01074-2. PMC12046608 39394525

[acel14471-bib-0025] Goodfellow, I. , Y. Bengio , and A. Courville . 2016. Deep Learning. Cambridge, MA: MIT Press.

[acel14471-bib-0026] Hafner, A. , M. L. Bulyk , A. Jambhekar , and G. Lahav . 2019. “The Multiple Mechanisms That Regulate p53 Activity and Cell Fate.” Nature Reviews Molecular Cell Biology 20, no. 4: 199–210. 30824861 10.1038/s41580-019-0110-x

[acel14471-bib-0027] Hannum, G. , J. Guinney , L. Zhao , et al. 2013. “Genome‐Wide Methylation Profiles Reveal Quantitative Views of Human Aging Rates.” Molecular Cell 49, no. 2: 359–367. 23177740 10.1016/j.molcel.2012.10.016 PMC3780611

[acel14471-bib-0028] Hartman, E. , A. M. Scott , C. Karlsson , et al. 2023. “Interpreting Biologically Informed Neural Networks for Enhanced Proteomic Biomarker Discovery and Pathway Analysis.” Nature Communications 14, no. 1: 5359. 10.1038/s41467-023-41146-4 PMC10475049 37660105

[acel14471-bib-0029] Horvath, S. 2013. “DNA Methylation Age of Human Tissues and Cell Types.” Genome Biology 14: 1–20. 10.1186/gb-2013-14-10-r115 PMC4015143 24138928

[acel14471-bib-0030] Jacobs, R. A. , M. I. Jordan , S. J. Nowlan , and G. E. Hinton . 1991. “Adaptive Mixtures of Local Experts.” Neural Computation 3, no. 1: 79–87. 31141872 10.1162/neco.1991.3.1.79

[acel14471-bib-0031] Jonsson, B. A. , G. Bjornsdottir , T. E. Thorgeirsson , et al. 2019. “Brain Age Prediction Using Deep Learning Uncovers Associated Sequence Variants.” Nature Communications 10, no. 1: 5409. 10.1038/s41467-019-13163-9 PMC6881321 31776335

[acel14471-bib-0032] Keskar, N. S. , D. Mudigere , J. Nocedal , M. Smelyanskiy , and P. T. Tang . 2017. “On Large‐Batch Training for Deep Learning: Generalization Gap and Sharp Minima” International Conference on Learning Representations.

[acel14471-bib-0033] Kingma, D. P. , and J. Ba . 2015. “Adam: A Method for Stochastic Optimization.” International Conference on Learning Representations. PMC13460018 42583277

[acel14471-bib-0034] Kitagishi, Y. , A. Nakanishi , Y. Ogura , and S. Matsuda . 2014. “Dietary Regulation of PI3K/AKT/GSK‐3β Pathway in Alzheimer's Disease.” Alzheimer's Research & Therapy 6: 1–7. 10.1186/alzrt265 PMC4075129 25031641

[acel14471-bib-0035] Korsunsky, I. , N. Millard , J. Fan , et al. 2019. “Fast, Sensitive and Accurate Integration of Single‐Cell Data With Harmony.” Nature Methods 16, no. 12: 1289–1296. 31740819 10.1038/s41592-019-0619-0 PMC6884693

[acel14471-bib-0036] Krizhevsky, A. , I. Sutskever , and G. E. Hinton . 2012. “ImageNet Classification With Deep Convolutional Neural Networks.” Advances in Neural Information Processing Systems 60: 84–90.

[acel14471-bib-0037] Krshnan, L. , M. L. van de Weijer , and P. Carvalho . 2022. “Endoplasmic Reticulum–Associated Protein Degradation.” Cold Spring Harbor Perspectives in Biology 14, no. 12: a041247. 35940909 10.1101/cshperspect.a041247 PMC9732900

[acel14471-bib-0038] Li, H. , C. R. Brouwer , and W. Luo . 2022. “A Universal Deep Neural Network for In‐Depth Cleaning of Single‐Cell RNA‐Seq Data.” Nature Communications 13, no. 1: 1901. 10.1038/s41467-022-29576-y PMC8990021 35393428

[acel14471-bib-0039] Li, R. , W. Chen , M. Li , et al. 2023. “LensAge Index as a Deep Learning‐Based Biological Age for Self‐Monitoring the Risks of Age‐Related Diseases and Mortality.” Nature Communications 14, no. 1: 7126. 10.1038/s41467-023-42934-8 PMC10628111 37932255

[acel14471-bib-0040] Li, S. , Y. Zhao , R. Varma , et al. 2020. “PyTorch Distributed: Experiences on Accelerating Data Parallel Training.” Proceedings of the VLDB Endowment 13, no. 12: 3005–3018.

[acel14471-bib-0041] Liu, J. , D. Zhang , D. Yu , M. Ren , and J. Xu . 2021. “Machine Learning Powered Ellipsometry.” Light: Science & Applications 10, no. 1: 55. 10.1038/s41377-021-00482-0 PMC7952555 33707413

[acel14471-bib-0042] Liu, X. , Z. Liu , Z. Wu , et al. 2023. “Resurrection of Endogenous Retroviruses During Aging Reinforces Senescence.” Cell 186, no. 2: 287–304. 36610399 10.1016/j.cell.2022.12.017

[acel14471-bib-0043] López‐Otín, C. , M. A. Blasco , L. Partridge , M. Serrano , and G. Kroemer . 2023. “Hallmarks of Aging: An Expanding Universe.” Cell 186, no. 2: 243–278. 36599349 10.1016/j.cell.2022.11.001

[acel14471-bib-0044] Lu, X. 2023. “Regulation of Endogenous Retroviruses in Murine Embryonic Stem Cells and Early Embryos.” Journal of Molecular Cell Biology 15, no. 8: mjad052. 10.1093/jmcb/mjad052 PMC10794949 37604781

[acel14471-bib-0045] Lundberg, S. M. , and S. I. Lee . 2017. “A Unified Approach to Interpreting Model Predictions.” Advances in Neural Information Processing Systems 30: 4765–4774.

[acel14471-bib-0046] Ma, J. , X. Xu , M. Li , et al. 2021. “Predictive Models of Aging of the Human Eye Based on Ocular Anterior Segment Morphology.” Journal of Biomedical Informatics 120: 103855. 34216803 10.1016/j.jbi.2021.103855

[acel14471-bib-0047] Manjón, J. V. , P. Coupé , and A. Buades . 2015. “MRI Noise Estimation and Denoising Using Non‐Local PCA.” Medical Image Analysis 22, no. 1: 35–47. 25725303 10.1016/j.media.2015.01.004

[acel14471-bib-0048] Mao, S. , J. Su , L. Wang , X. C. Bo , C. Li , and H. Chen . 2023. “A Transcriptome‐Based Single‐Cell Biological Age Model and Resource for Tissue‐Specific Aging Measures.” Genome Research 33, no. 8: 1381–1394. 37524436 10.1101/gr.277491.122 PMC10547252

[acel14471-bib-0049] Meyer, D. H. , and B. Schumacher . 2021. “BiT Age: A Transcriptome‐Based Aging Clock Near the Theoretical Limit of Accuracy.” Aging Cell 20, no. 3: e13320. 33656257 10.1111/acel.13320 PMC7963339

[acel14471-bib-0050] Mishra, S. , I. Beheshti , and P. Khanna . 2023. “A Review of Neuroimaging‐Driven Brain Age Estimation for Identification of Brain Disorders and Health Conditions.” IEEE Reviews in Biomedical Engineering 16: 371–385. 34428153 10.1109/RBME.2021.3107372

[acel14471-bib-0051] Mohamadi, S. , and D. A. Adjeroh . 2021. “An Information‐Theoretic Framework for Identifying Age‐Related Genes Using Human Dermal Fibroblast Transcriptome Data.” In IEEE International Conference on Bioinformatics and Biomedicine, 2294–2300. Cambridge, MA: IEEE.

[acel14471-bib-0052] Mohamadi, S. , N. M. Nasrabadi , G. Doretto , and D. A. Adjeroh . 2021. “Human Age Estimation From Gene Expression Data Using Artificial Neural Networks.” In IEEE International Conference on Bioinformatics and Biomedicine, 3492–3497. Cambridge, MA: IEEE.

[acel14471-bib-0053] Moutsopoulos, I. , L. Maischak , E. Lauzikaite , et al. 2021. “noisyR: Enhancing Biological Signal in Sequencing Datasets by Characterizing Random Technical Noise.” Nucleic Acids Research 49, no. 14: e83. 34076236 10.1093/nar/gkab433 PMC8373073

[acel14471-bib-0054] Nikoletopoulou, V. , E. Kyriakakis , and N. Tavernarakis . 2014. “Cellular and Molecular Longevity Pathways: The Old and the New.” Trends in Endocrinology and Metabolism 25, no. 4: 212–223. 24388148 10.1016/j.tem.2013.12.003

[acel14471-bib-0055] Noothout, J. M. , B. D. De Vos , J. M. Wolterink , et al. 2022. “Deep Learning‐Based Regression and Classification for Automatic Landmark Localization in Medical Images.” IEEE Transactions on Medical Imaging 39, no. 12: 4011–4022. 10.1109/TMI.2020.3009002 32746142

[acel14471-bib-0056] Pappireddi, N. , L. Martin , and M. Wühr . 2019. “A Review on Quantitative Multiplexed Proteomics.” Chembiochem 20, no. 10: 1210–1224. 30609196 10.1002/cbic.201800650 PMC6520187

[acel14471-bib-0057] Paszke, A. , S. Gross , F. Massa , et al. 2019. “PyTorch: An Imperative Style, High‐Performance Deep Learning Library.” Advances in Neural Information Processing Systems 32: 8024–8035.

[acel14471-bib-0058] Pedregosa, F. , G. Varoquaux , A. Gramfort , et al. 2011. “Scikit‐Learn: Machine Learning in Python.” Journal of Machine Learning Research 12: 2825–2830.

[acel14471-bib-0059] Qi, H. , Y. Wu , W. Zhang , N. Yu , X. Lu , and J. Liu . 2024. “The Syntaxin‐Binding Protein STXBP5 Regulates Progerin Expression.” Scientific Reports 14, no. 1: 23376. 39379476 10.1038/s41598-024-74621-z PMC11461833

[acel14471-bib-0060] Reya, T. , S. J. Morrison , M. F. Clarke , and I. L. Weissman . 2011. “Stem Cells, Cancer, and Cancer Stem Cells.” Nature 414, no. 6859: 105–111. 10.1038/35102167 11689955

[acel14471-bib-0061] Rutledge, J. , H. Oh , and T. Wyss‐Coray . 2022. “Measuring Biological Age Using Omics Data.” Nature Reviews Genetics 23, no. 12: 715–727. 10.1038/s41576-022-00511-7 PMC10048602 35715611

[acel14471-bib-0062] Salehinejad, H. , E. Colak , T. Dowdell , J. Barfett , and S. Valaee . 2018. “Synthesizing Chest X‐Ray Pathology for Training Deep Convolutional Neural Networks.” IEEE Transactions on Medical Imaging 38, no. 5: 1197–1206. 30442603 10.1109/TMI.2018.2881415

[acel14471-bib-0063] Savcisens, G. , T. Eliassi‐Rad , L. K. Hansen , et al. 2023. “Using Sequences of Life‐Events to Predict Human Lives.” Nature Computational Science 4, no. 1: 43–56. 38177491 10.1038/s43588-023-00573-5

[acel14471-bib-0064] Selvaraju, R. R. , M. Cogswell , A. Das , R. Vedantam , D. Parikh , and D. Batra . 2017. “Grad‐Cam: Visual Explanations From Deep Networks via Gradient‐Based Localization.” In Proceedings of the IEEE International Conference on Computer Vision, 618–626. Cambridge, MA: IEEE.

[acel14471-bib-0065] Sherman, B. T. , M. Hao , J. Qiu , et al. 2022. “DAVID: A Web Server for Functional Enrichment Analysis and Functional Annotation of Gene Lists (2021 Update).” Nucleic Acids Research 50, no. W1: W216–W221. 35325185 10.1093/nar/gkac194 PMC9252805

[acel14471-bib-0066] Shokhirev, M. N. , and A. A. Johnson . 2021. “Modeling the Human Aging Transcriptome Across Tissues, Health Status, and Sex.” Aging Cell 20, no. 1: e13280. 33336875 10.1111/acel.13280 PMC7811842

[acel14471-bib-0067] Shwartz‐Ziv, R. , and A. Armon . 2022. “Tabular Data: Deep Learning Is Not all You Need.” Information Fusion 81: 84–90.

[acel14471-bib-0068] Srivastava, N. , G. Hinton , A. Krizhevsky , I. Sutskever , and R. Salakhutdinov . 2014. “Dropout: A Simple Way to Prevent Neural Networks From Overfitting.” Journal of Machine Learning Research 15, no. 1: 1929–1958.

[acel14471-bib-0069] Suhre, K. , M. I. McCarthy , and J. M. Schwenk . 2021. “Genetics Meets Proteomics: Perspectives for Large Population‐Based Studies.” Nature Reviews Genetics 22, no. 1: 19–37. 10.1038/s41576-020-0268-2 32860016

[acel14471-bib-0070] Suryadevara, V. , A. D. Hudgins , A. Rajesh , et al. 2024. “SenNet Recommendations for Detecting Senescent Cells in Different Tissues.” Nature Reviews Molecular Cell Biology 25: 1–23. 10.1038/s41580-024-00738-8 PMC11578798 38831121

[acel14471-bib-0071] Szklarczyk, D. , R. Kirsch , M. Koutrouli , et al. 2023. “The STRING Database in 2023: Protein–Protein Association Networks and Functional Enrichment Analyses for any Sequenced Genome of Interest.” Nucleic Acids Research 51, no. D1: D638–D646. 36370105 10.1093/nar/gkac1000 PMC9825434

[acel14471-bib-0072] Wu, Y. , J. Liu , Y. Wang , S. Gibson , M. Osadchy , and Y. Fang . 2024. “Reconstructing Randomly Masked Spectra Helps DNNs Identify Discriminant Wavenumbers.” IEEE Transactions on Pattern Analysis and Machine Intelligence 46, no. 5: 3845–3861. 38150338 10.1109/TPAMI.2023.3347617

[acel14471-bib-0073] Xu, H. , C. Caramanis , and S. Mannor . 2009. “Robustness and Regularization of Support Vector Machines.” Journal of Machine Learning Research 10, no. 7: 1485–1510.

[acel14471-bib-0074] Yuksel, S. E. , J. N. Wilson , and P. D. Gader . 2012. “Twenty Years of Mixture of Experts.” IEEE Transactions on Neural Networks and Learning Systems 23, no. 8: 1177–1193. 24807516 10.1109/TNNLS.2012.2200299

[acel14471-bib-0075] Zhang, L. , X. Wang , D. Yang , et al. 2020. “Generalizing Deep Learning for Medical Image Segmentation to Unseen Domains via Deep Stacked Transformation.” IEEE Transactions on Medical Imaging 39, no. 7: 2531–2540. 32070947 10.1109/TMI.2020.2973595 PMC7393676

[acel14471-bib-0076] Zhang, Y. , R. Wilson , J. Heiss , et al. 2017. “DNA Methylation Signatures in Peripheral Blood Strongly Predict All‐Cause Mortality.” Nature Communications 8, no. 1: 14617. 10.1038/ncomms14617 PMC5357865 28303888

[acel14471-bib-0077] Zhao, Y. , H. Cai , Z. Zhang , J. Tang , and Y. Li . 2021. “Learning Interpretable Cellular and Gene Signature Embeddings From Single‐Cell Transcriptomic Data.” Nature Communications 12, no. 1: 5261. 10.1038/s41467-021-25534-2 PMC8421403 34489404

