# Supplementary material for: DeepQA: A Unified Transcriptome‐Based Aging Clock Using Deep Neural Networks
Source: Aging Cell. 2025 Jan 5;24(5):e14471. doi: 10.1111/acel.14471 (PMC12074024; doi:10.1111/acel.14471)
Supplement: Supplementary file 1 — Data S1. [file ACEL-24-e14471-s002.pdf]

Supplementary Information for

"DeepQA: a unified transcriptome-based aging clock using deep neural networks "

Hongqian Qi, Hongchen Zhao, Enyi Li, Xinyi Lu, Ningbo Yu, Jinchao Liu, Jianda Han

S1. Evaluation of prediction performance of aging clocks on unhealthy subjects

We followed the protocol proposed in the reference [1], the details of which is as follows: First compute $PAD = Y_{pred} - Y\_c$, which is then adjusted for age, batch and gender; Then, calculate $PAD\_Difference = Mean(PAD\_unhealthy) - Mean(PAD\_control)$; Finally perform t-test followed by Bonferroni correction.

[1] Jonsson, B. A., Bjornsdottir, G., Thorgeirsson, T. E., Ellingsen, L. M., Walters, G. B., Gudbjartsson, D. F., Stefansson, H., Stefansson, K. and Ulfarsson, M. O. (2019) Brain age prediction using deep learning uncovers associated sequence variants. *Nature Communications*, 10, 5409.

S2. Results of competing methods on the database MCATS

Table S1 DeepQA (Trained on both healthy and unhealthy subjects)

Group	PAD Difference	95% CI	Corrected p-value	Significance	# Cases	# Controls
AD	3.836	(2.469, 5.202)	4.79E-07	TRUE	119	261
AMD	1.107	(0.343, 1.870)	2.29E-02	TRUE	392	494
Schizophrenia	1.589	(0.531, 2.646)	2.12E-02	TRUE	57	469
DCM	-0.559	(-1.670, 0.553)	1.00E+00	FALSE	162	821
Dysplasia	2.105	(0.342, 3.868)	1.14E-01	FALSE	50	500

Table S2 EN-AllGenes (Trained on healthy subjects)

Group	PAD Difference	95% CI	Corrected p-value	Significance	# Cases	# Controls
AD	-0.306	(-1.838,1.226)	1.00E+00	FALSE	119	261
AMD	-0.842	(-1.739,0.055)	3.30E-01	FALSE	392	494
Schizophrenia	9.449	(7.021,11.877)	9.69E-10	TRUE	57	469
DCM	6.458	(5.242, 7.674)	4.24E-20	TRUE	162	821
Dysplasia	8.282	(5.930,10.634)	2.62E-08	TRUE	50	500

Table S3 EN-Variable (Trained on healthy subjects)

Group	PAD Difference	95% CI	Corrected p-value	Significance	# Cases	# Controls
AD	-0.338	(-2.001,1.325)	1.00E+00	FALSE	119	261
AMD	-0.586	(-1.569,0.397)	1.00E+00	FALSE	392	494
Schizophrenia	10.675	(7.809, 3.542)	3.73E-09	TRUE	57	469
DCM	5.024	(3.786, 6.261)	3.89E-13	TRUE	162	821
Dysplasia	7.885	(5.603, 0.167)	3.46E-08	TRUE	50	500

Table S4 EN-Differential (Trained on healthy subjects)

Group	PAD Difference	95% CI	Corrected p-value	Significance	# Cases	# Controls
AD	3.892	(2.345, 5.438)	7.30E-06	TRUE	119	261
AMD	-1.258	(-2.345,-0.171)	1.18E-01	FALSE	392	494
Schizophrenia	8.262	(5.636, 10.888)	2.82E-07	TRUE	57	469
DCM	6.215	(4.825, 7.604)	2.11E-15	TRUE	162	821
Dysplasia	8.550	(6.196, 10.904)	8.30E-09	TRUE	50	500

Table S5 EN-AgingMap (Trained on healthy subjects)

Group	PAD Difference	95% CI	Corrected p-value	Significance	# Cases	# Controls
AD	-0.842	(-2.292, 0.609)	1.00E+00	FALSE	119	261
AMD	-0.734	(-1.756, 0.289)	8.00E-01	FALSE	392	494
Schizophrenia	8.919	(6.337, 11.5)	2.48E-08	TRUE	57	469
DCM	5.546	(4.010, 7.081)	1.08E-10	TRUE	162	821
Dysplasia	4.930	(2.624, 7.235)	4.75E-04	TRUE	50	500

Table S6 RF-Variable (Trained on healthy subjects)

Group	PAD Difference	95% CI	Corrected p-value	Significance	# Cases	# Controls
AD	-1.700	(-3.471,0.072)	3.08E-01	FALSE	119	261
AMD	-1.238	(-2.304,-0.172)	1.15E-01	FALSE	392	494
Schizophrenia	10.121	(7.654, 12.589)	1.78E-10	TRUE	57	469
DCM	2.690	(1.124, 4.255)	4.57E-03	TRUE	162	821
Dysplasia	5.426	(3.127, 7.726)	1.09E-04	TRUE	50	500

Table S7 RF-Differential (Trained on healthy subjects)

Group	PAD Difference	95% CI	Corrected p-value	Significance	# Cases	# Controls
AD	-1.417	(-3.283, 0.45)	6.92E-01	FALSE	119	261
AMD	-1.155	(-2.231,-0.079)	1.78E-01	FALSE	392	494
Schizophrenia	9.495	(6.972, 12.017)	2.22E-09	TRUE	57	469
DCM	2.509	(0.930, 4.089)	1.06E-02	TRUE	162	821
Dysplasia	5.270	(2.998, 7.543)	1.38E-04	TRUE	50	500

Table S8 CNN2D (Trained on healthy subjects)

Group	PAD Difference	95% CI	Corrected p-value	Significance	# Cases	# Controls
AD	-4.088	(-5.641,-2.535)	2.69E-06	TRUE	119	261
AMD	-4.568	(-5.676,-3.461)	1.16E-14	TRUE	392	494
Schizophrenia	6.189	(3.877, 8.501)	9.01E-06	TRUE	57	469
DCM	6.112	(4.511, 7.712)	1.16E-11	TRUE	162	821
Dysplasia	2.424	(0.162, 4.687)	2.01E-01	FALSE	50	500

Table S9 ELDA-GEF (Trained on healthy subjects)

Group	PAD Difference	95% CI	Corrected p-value	Significance	# Cases	# Controls
AD	1.154	(-0.571, 2.878)	9.56E-01	FALSE	119	261
AMD	-0.559	(-1.469, 0.351)	1.00E+00	FALSE	392	494
Schizophrenia	9.063	(7.073, 11.053)	3.17E-12	TRUE	57	469
DCM	9.610	(8.184, 11.037)	2.53E-28	TRUE	162	821
Dysplasia	4.478	(2.270, 6.686)	1.00E-03	TRUE	50	500

Table S10 RF-Variable (Trained on healthy subjects of corresponding cohorts/organs)

Group	PAD Difference	95% CI	Corrected p-value	Significance	# Cases	# Controls
AD	2.319	(-0.475, 5.113)	5.39E-01	FALSE	85	41
AMD	-5.071	(-6.602, -3.539)	1.66E-09	TRUE	350	172
Schizophrenia	5.753	(1.625, 9.881)	4.74E-02	TRUE	26	32
DCM	0.900	(-1.076, 2.876)	1.00E+00	FALSE	157	121
Dysplasia	0.083	(-2.689, 2.855)	1.00E+00	FALSE	33	24

Table S11 RF- Differential (Trained on healthy subjects of corresponding cohorts/organs)

Group	PAD Difference	95% CI	Corrected p-value	Significance	# Cases	# Controls
AD	-0.492	(-3.333, 2.349)	1.00E+00	FALSE	85	41
AMD	-4.867	(-6.408, -3.327)	8.91E-09	TRUE	350	172
Schizophrenia	5.265	(1.508, 9.022)	4.37E-02	TRUE	26	32
DCM	-0.790	(-2.810, 1.229)	1.00E+00	FALSE	157	121
Dysplasia	-0.404	(-3.672, 2.864)	1.00E+00	FALSE	33	24

S4. Implementation details of generating PCA noises

Formally, any (normalized) sample of gene expression data can be decomposed into a linear weighted combination of principal components (PCs), which are learned from the data.

$$x = \sum_{k=0}^{N-1} \mu_k \, \mathrm{PC}_k = \sum_{k=0}^{L-1} \alpha_k \, \mathrm{PC}_k + \sum_{k=L}^{N-1} \beta_k \, \mathrm{PC}_k$$

where the first L terms correspond to PCs contributed to 95% of the variances of the data. The rest of the terms $(N - L)$ in total contributed to 5% of the variances which are often regarded as noises. This allows us to simulate inherent noises by manipulating the weights of these “noisy” PCs, i.e.

$$\tilde{x} = \sum_{k=0}^{L-1} \alpha_k \, \mathrm{PC}_k + \sum_{k=L}^{N-1} \rho \cdot \beta_k \, \mathrm{PC}_k$$

where $\rho > 0$ and $\tilde{x}$ is the noisy version of the original sample x. As PCA is an unsupervised learning method and no test set leakage would occur.

We adopted the PCA implementation of the scikit-learn machine learning library and have used the default hyperparameters for the PCA function including *whiten=False, svd_solver='auto', tol=0.0, iterated_power='auto', n_oversamples=10, power_iteration_normalizer='auto'.*

Table S13. Some details of PCs used for generating PCA noises for each of the compared methods. Explained variances show that the PCs for generating PCA noises contributed to 5% of the total variances in gene expression data.

Methods	# PCs for PCA noises	# PCs in total	Explained variances
DeepQA	12903	13388	5%
EN-All	12903	13388	5%
EN-Var	812	1000	5%
EN-Diff	889	998	5%
EN-Agingmap	504	646	5%
ELDA-GEF	12903	13388	5%
RF-Var	812	1000	5%
RF-Diff	889	998	5%
CNN-2D	12903	13388	5%

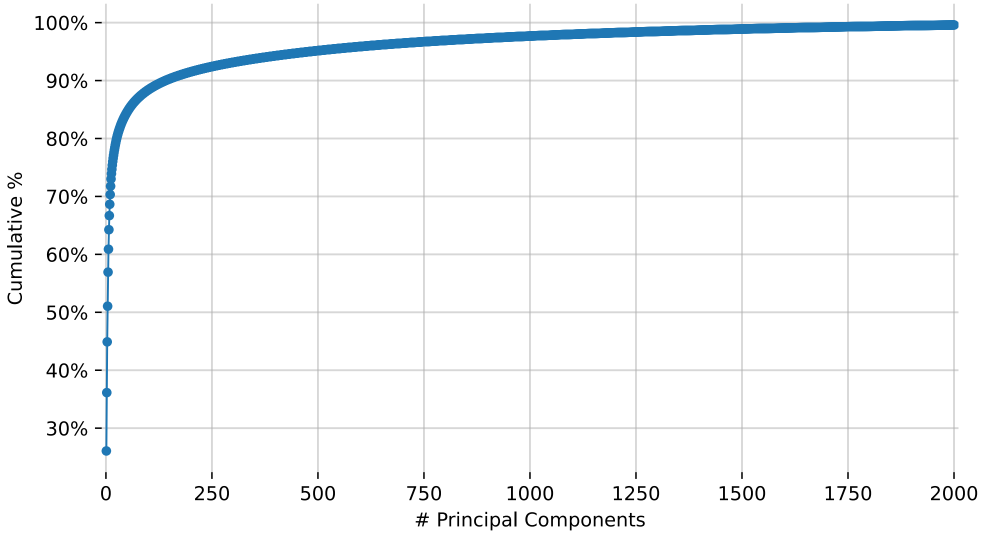

Figure S1. Explained variances v.s. number of principal components in the experiment of generating PCA noises for DeepQA. In the experiments of robustness test, we applied Principal Component Analysis (PCA) to find factors which contribute less to the explained variances in the data. All the PCs corresponding to 95% - 100% of explained variances were used to generate noises.

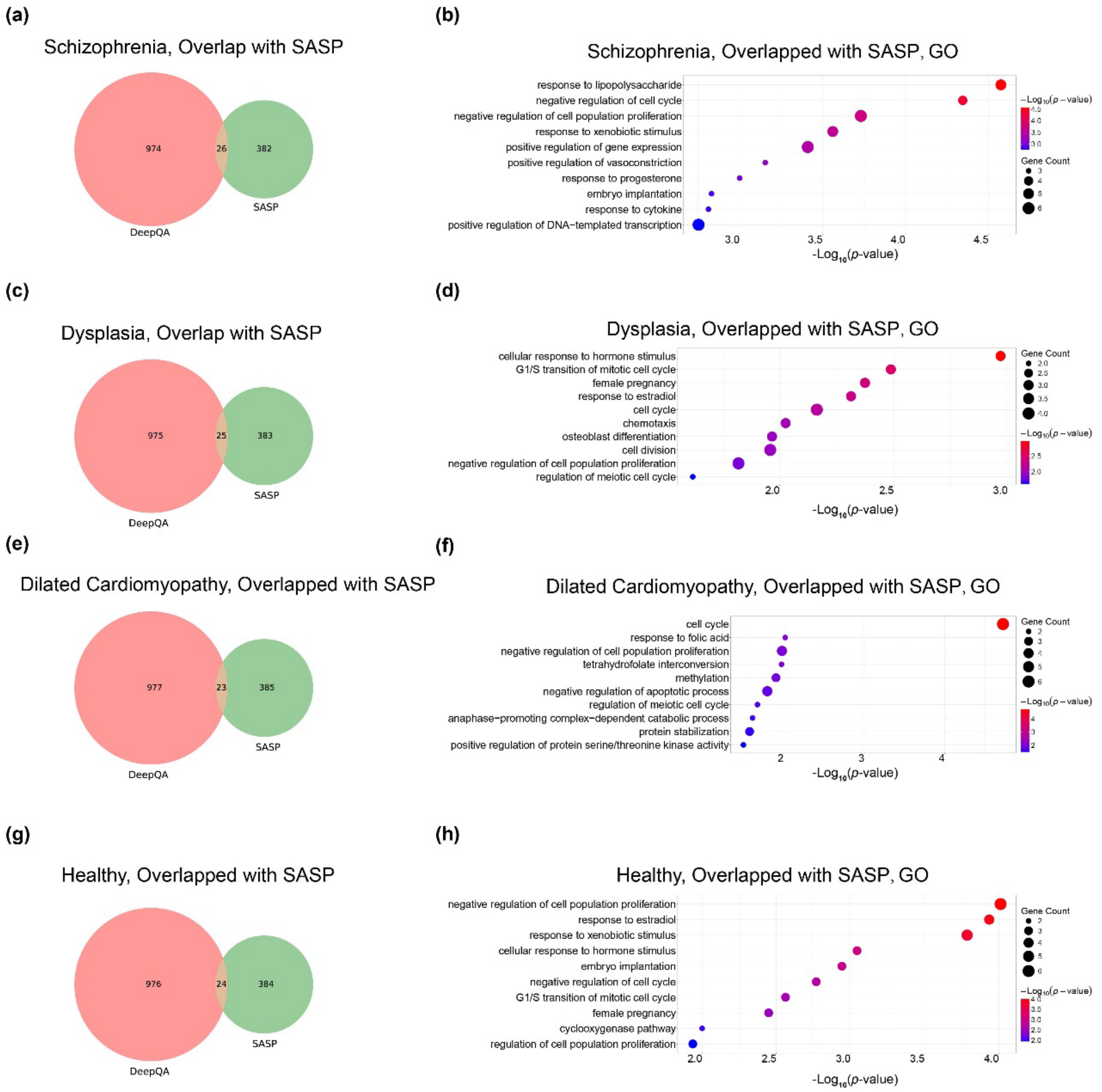

Figure S2. (a,c,e,g) Overlap between genes identified by DeepQA and SASP genes, on samples with conditions of Schizophrenia, Dysplasia, Dilated Cardiomyopathy and Healthy respectively. (b,d,f,h) Functional analysis of overlapped genes corresponding to samples with conditions of Schizophrenia, Dysplasia, Dilated Cardiomyopathy and Healthy respectively.
